# Associations of bacterial enteropathogens with systemic inflammation, iron deficiency, and anemia in preschool-age children in southern Ghana

**DOI:** 10.1371/journal.pone.0271099

**Published:** 2022-07-08

**Authors:** Nathalie J. Lambrecht, Dave Bridges, Mark L. Wilson, Bright Adu, Joseph N. S. Eisenberg, Gloria Folson, Ana Baylin, Andrew D. Jones

**Affiliations:** 1 Department of Nutritional Sciences, School of Public Health, University of Michigan, Ann Arbor, Michigan, United States of America; 2 Institute of Public Health, Charité, Universitätsmedizin Berlin, Corporate Member of Freie Universität Berlin and Humboldt-Universität zu Berlin, Berlin, Germany; 3 Research Department 2, Potsdam Institute for Climate Impact Research (PIK), Member of the Leibniz Association, Potsdam, Germany; 4 Department of Epidemiology, School of Public Health, University of Michigan, Ann Arbor, Michigan, United States of America; 5 Department of Immunology, Noguchi Memorial Institute for Medical Research, University of Ghana, Legon, Ghana; 6 Department of Nutrition, Noguchi Memorial Institute for Medical Research, University of Ghana, Legon, Ghana; Njala University, SIERRA LEONE

## Abstract

Anemia remains a pervasive public health problem among preschool-age children in Ghana. Recent analyses have found that anemia in Ghanaian children, particularly in Southern regions, is largely attributable to infectious causes, rather than nutritional factors. Infections with enteropathogens can reduce iron absorption and increase systemic inflammation, but few studies have examined direct links between enteropathogens and anemia. This study investigated associations between detection of individual bacterial enteropathogens and systemic inflammation, iron deficiency, and anemia among 6- to 59-month-old children in Greater Accra, Ghana. Serum samples were analyzed from a cross-sectional sample of 262 children for concentrations of hemoglobin (Hb), biomarkers of systemic inflammation [C-reactive protein (CRP) and α-1-acid glycoprotein (AGP)], and biomarkers of iron status [serum ferritin (SF) and serum transferrin receptor (sTfR)]. Stool samples were analyzed for ten bacterial enteropathogens using qPCR. We estimated associations between presence of each enteropathogen and elevated systemic inflammation (CRP > 5 mg/L and AGP > 1 g/L), iron deficiency (SF < 12 μg/L and sTfR > 8.3 mg/L) and anemia (Hb < 110 g/L). Enteropathogens were detected in 87% of children’s stool despite a low prevalence of diarrhea (6.5%). Almost half (46%) of children had anemia while one-quarter (24%) had iron deficiency (low SF). Despite finding no associations with illness symptoms, *Campylobacter jejuni/coli* detection was strongly associated with elevated CRP [Odds Ratio (95% CI): 3.49 (1.45, 8.41)] and elevated AGP [4.27 (1.85, 9.84)]. Of the pathogens examined, only enteroinvasive *Escherichia coli/Shigella* spp. (EIEC/*Shigella*) was associated with iron deficiency, and enteroaggregative *Escherichia coli* (EAEC) [1.69 (1.01, 2.84)] and EIEC/*Shigella* [2.34 (1.15, 4.76)] were associated with anemia. These results suggest that certain enteroinvasive pathogenic bacteria may contribute to child anemia. Reducing exposure to enteropathogens through improved water, sanitation, and hygiene practices may help reduce the burden of anemia in young Ghanaian children.

## Introduction

In Ghana, anemia affects 60% of children aged 6 to 59 months [1], with regional burdens up to 82% [2]. Anemia involves inadequate oxygen delivery to tissues due to insufficient red blood cells or functional hemoglobin, putting children at risk of impaired cognitive and motor development, as well as increased mortality [3–5]. Various factors can lead to anemia including inadequate intake or absorption of hematopoietic nutrients, infectious diseases and inflammation, and genetic hemoglobin disorders [6, 7]. While iron deficiency is the primary cause of anemia in preschool-age children globally [8], the proportion of anemia attributable to iron deficiency varies by context, including infectious disease burdens [9]. Analysis of the 2017 Ghana Micronutrient Survey found that only 35% of total anemia in 6-59-month-old children was associated with iron deficiency [10]. In the Southern Belt of Ghana, where our study took place, anemia was neither associated with children’s iron status nor vitamin A deficiency [11]. Indeed, three-quarters of the anemia burden was attributable to factors associated with infectious diseases (i.e., malaria, inflammation, and fever) [11]. To reduce the anemia burden, understanding the causes of inflammation, especially from non-malarial infections, is thus critical [12, 13]. An emerging, but understudied hypothesis is that exposure to enteropathogens directly contributes to anemia in children [14].

Recurrent infection with a range of protozoal, viral, and bacterial enteropathogens is common among young children in low- and middle-income countries (LMICs), even among children asymptomatic for diarrhea [15, 16]. Studies have linked subclinical enteric pathogen carriage to linear growth faltering and cognitive development delays, which are posited to occur, in part, through a condition of gastrointestinal inflammation and permeability known as environmental enteric dysfunction (EED) [17–23]. There are two plausible mechanisms by which enteropathogen infections may also lead to iron deficiency and anemia. First, persistent infection with enteropathogens can lead to morphological changes of the intestinal villi (a characteristic of EED) which reduces absorption of iron and other hematopoietic nutrients [24, 25]. Second, enteropathogen infections may promote a systemic inflammatory response which induces iron sequestration and reduces intestinal iron absorption via hepcidin-mediated signaling [7, 26, 27]. Over time, this “functional iron deficiency”–in which the body has sufficient iron stores but iron is not available to tissues–can lead to reduced erythropoiesis and anemia [12].

Observational studies have identified positive associations between EED biomarkers and anemia, supporting a link between enteropathogen exposure and anemia [28–31]. A few studies that have measured enteropathogens provide further evidence for this association. A longitudinal birth cohort study conducted in eight LMICs found that higher rates of enteropathogen detection in stool over children’s first two years of life was associated with lower hemoglobin concentrations [23]. Analyses of the baseline microbiome of anemic school-age children in Côte d’Ivoire and anemic 6-month-old infants in Kenya enrolled in iron fortification trials revealed an adverse abundance of pathogenic enterobacteria and lower ratio of commensal bacteria when compared to non-anemic children [32, 33]. Among the school-age children, however, no correlations between the number of fecal enterobacteria and baseline iron biomarker concentrations were observed [32]. Building on this body of literature, our study is the first to examine pathogen-specific associations with iron status and anemia among African preschool-age children.

In this study, we assessed the associations between detection of specific bacterial enteropathogens and elevated inflammation, iron deficiency, and anemia in children 6–59 months old in Greater Accra, Ghana. We hypothesized that detection of enteropathogens would be associated with higher odds of systemic inflammation, iron deficiency, and anemia. We did not have *a priori* hypotheses about the independent effect of bacterial pathogens on iron status and anemia, and thus analyzed each pathogen with all the outcomes of interest.

## Methods

### Study design

This cross-sectional study analyzed a subsample of 6–59-month-old children from a study conducted in the Greater Accra region, Ghana investigating livestock ownership and anemia [34]. Overall, 484 households from 18 semi-rural communities in the Ga East and Shai Osudoku districts were sampled between October and November 2018. Communities ranged in population size from small (<750 residents) to large (>2,000 residents). From 13 to 40 households were sampled per community, depending on community size, each with at least one child aged 6–59 months old (the index child). In households with more than one child in the target age range, the youngest child was recruited to participate. To be eligible for inclusion, the index child’s primary caregiver, usually their mother, had to be at least 18 years old.

**Fig 1:**
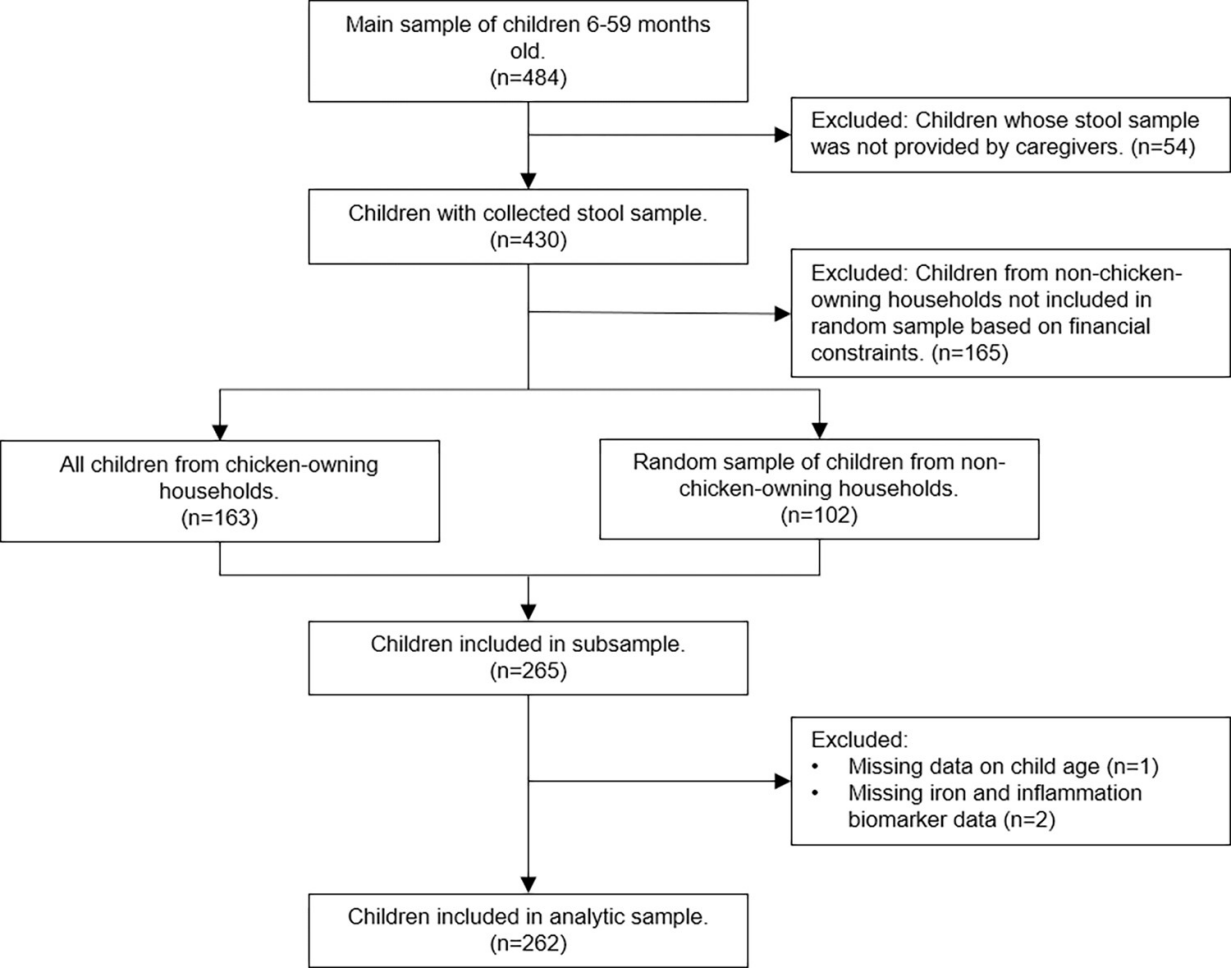
Flow chart describing sample selection.

From the sample of 484 children, 430 (89%) had available stool samples, while stool samples were unable to be obtained from 54 children (**Fig 1**). Of those with a stool sample, a subsample of 265 children (62%) was selected for analysis due to the financial constraints of conducting enteropathogen testing. Based on an associated study investigating whether chicken ownership and detection of pathogenic bacteria in chickens’ feces was associated with enteropathogen infection in children [35], this 265-child subsample comprised all 163 children from chicken-owning households and 102 of 267 children (38%) from a random sample of non-chicken owning households. Children included in the subsample were not significantly different in sex or age from those who were excluded.

### Survey data collection

Trained enumerators interviewed each index child’s primary caregiver, recording data using electronic tablets (Samsung Galaxy Tab A, Model Number SM-T285) with the Qualtrics survey platform (Qualtrics, Provo, UT, USA). Data were collected on the household’s sociodemographic characteristics and the index child’s age and health. The child’s date of birth was verified using their child health record. The caregiver was asked whether the child experienced any symptoms of illness in the preceding seven days [i.e., diarrhea (≥3 loose stools in 24 hours) with or without blood, fever, illness with a cough, vomiting, or nausea].

### Blood sample collection and analysis

Child capillary blood samples were collected using a finger-prick [36]. After wiping away the first drop of blood, hemoglobin concentration (Hb) was measured using a HemoCue^®^ Hb 201+ portable hemoglobinometer (HemoCue AB, Ängelholm, Sweden) and malaria parasitemia was measured using the SD Malaria Ag P.f (HRP2/pLDH) antigen rapid diagnostic test (RDT) (Standard Diagnostics Inc., Gyeonggi‐do, Republic of Korea). Subsequently, 200 μL of capillary blood was collected in a Microvette^®^ clotting activator/serum tube (Sarstedt, Nümbrecht, Germany). Blood samples were transported at room temperature within five hours of collection to the Noguchi Memorial Institute for Medical Research in Accra, Ghana, spun at 5,000 rpm for 10 minutes, and then 50 μL of serum was aliquoted into 0.2 mL PCR tubes (Sarstedt, Nümbrecht, Germany). Serum samples were stored at ˗20°C until shipment on dry ice to the VitMin Laboratory in Willstaett, Germany, for biomarker analysis.

Serum samples were analyzed for serum ferritin (SF), serum transferrin receptor (sTfR), C-reactive protein (CRP), and α-1-acid glycoprotein (AGP) using a sandwich enzyme-linked immunosorbent assay (ELISA) [37]. SF and sTfR concentrations were adjusted for inflammation using the regression correction approach developed by the Biomarkers Reflecting Inflammation and Nutritional Determinants of Anemia (BRINDA) project [38]. Biomarker cut-offs for iron deficiency (SF and sTfR) and inflammation (CRP and AGP) were defined per the BRINDA project cut-offs for preschool-age children: inflammation-adjusted SF < 12 μg/L, inflammation-adjusted sTfR > 8.3 mg/L, CRP > 5 mg/L, and AGP > 1 g/L [38].

### Stool sample collection and analysis

Caregivers were instructed to collect their child’s morning stool in a sterile fecal container (Sarstedt, Nümbrecht, Germany) and store it in a shady spot until collected by the field team. Caregivers of children under 24 months old were provided with a diaper and those with older children a clean piece of paper on which the child should defecate to prevent environmental cross-contamination. Stool samples were collected each morning by the study team, stored in a cooler box with ice packs, and then transported to the Noguchi Memorial Institute for Medical Research for processing. If stool samples were not available or the child’s stool sample was improperly collected or stored, caregivers were provided with new stool collection supplies and contacted in the following days so that the field team could collect a new sample. Stool samples were analyzed for eggs of the soil-transmitted helminths *Ascaris lumbricoides*, *Trichuris trichiura*, *Ancylostoma duodenale*, and *Necator americanus* using Kato Katz methods [39]. Following duplicate thick smear preparation of each stool sample, two laboratory technicians independently analyzed the slides by microscopy for helminth eggs. For later enteropathogen analysis, unprocessed stool samples were aliquoted into 2 mL cryovials (Corning, Corning, NY, USA) using sterile techniques and stored at ˗80°C.

Microbial nucleic acid was extracted from stool samples using the QIAamp® PowerFecal® DNA Kit (Qiagen, Hilden, Germany). Following brief thawing, ~250 mg of stool was weighed and placed in a bead beating tube under a sterile hood. All extraction steps were then followed according to the kit protocol. In the final extraction step, 100 μL of DNA was eluted into an Eppendorf™ DNA LoBind microcentrifuge tube (Eppendorf, Hamburg, Germany). Each batch of nucleic acid extractions included an extraction blank to control for laboratory contamination, which went through all the extraction steps but without any addition of stool sample. Total DNA concentration and purity were measured using a NanoDrop™ 2000 spectrophotometer (ThermoFisher Scientific, Waltham, MA, USA). DNA samples were transported to the University of Michigan (Ann Arbor, MI, USA) and stored at ˗80°C until further analysis.

Taqman probe-based quantitative polymerase chain reaction (qPCR) was used to analyze DNA samples for the following bacterial enteropathogens: *Campylobacter jejuni*/*Campylobacter coli* (*C*. *jejuni/coli*), enteroaggregative *Escherichia (E*.*) coli* (EAEC), atypical enteropathogenic *E*. *coli* (aEPEC), typical enteropathogenic *E*. *coli* (tEPEC), heat-stable enterotoxin-producing *E*. *coli* (ST-ETEC), heat-labile enterotoxin-producing *E*. *coli* (LT-ETEC), *Salmonella enterica*, Shiga toxin-producing *E*. *coli* (STEC), enteroinvasive *E*. *coli/Shigella* species (EIEC/*Shigella*), and *Vibrio cholerae* (*V*. *cholerae*). Primer and probe sequences to identify pathogen gene targets were derived from Liu et al. [40] and Taniuchi et al. [41] (**S1 Table**). Based on aims of a prior study, the analyzed enteropathogens were identified as those which are most likely to be transmissible from poultry to humans and cause a substantial human disease burden [42]. We also analyzed enteropathogens associated with diarrhea or growth stunting in children [43, 44].

DNA samples and extraction blanks were run single-plex for each gene target on 384-well plates using the QuantStudio™ 5 System (Applied Biosystems™, Foster City, CA, USA). DNA samples were diluted 1:10 in ddH_2_O and run in triplicate or quadruplicate. Each plate also included a water control (ddH_2_O in place of DNA). Each amplification well contained 4.5 μL 1:10-diluted DNA sample, 5.0 μL TaqMan™ Fast Advanced Master Mix (Applied Biosystems™, Foster City, CA, USA), and 0.5 μL of primer-probe mixture at a final concentration of 500:250 nM primer:probe. Samples went through the following cycling conditions: 95°C for 10 minutes followed by 45 amplification cycles of 95°C for 15 s and 60°C for 1 min. For the *cadF* amplicon, annealing and extension were at 58°C for 1 min. Cycle threshold (Ct), which is inversely associated with pathogen load, was determined for each sample replicate using the Thermo Fisher Connect Design and Analysis Software Version 2.5 (Thermo Fisher Scientific, Carlsbad, CA, USA). The threshold line for each gene target was manually set to ensure comparability between plates by including a repeated positive sample on each plate. Sample technical replicates were inspected if the standard deviation between replicates was ≥ 0.5, and when warranted, technical outliers were manually removed. For each target gene, the sample Ct value was calculated as the average of amplified replicates. Samples with amplification of ≤ 50% of replicates were classified as negative. None of the extraction blanks or water controls amplified. A Ct cut-off of ≤ 35 was applied to define positive detection of a target gene. This cut-off was chosen as the lower bound at which we observed greater variability in Ct and incongruent amplification between technical replicates.

Positivity for enteropathogens was defined by presence of gene targets as follows: tEPEC (*eae* with *bfpA*, and without *stx1* or *stx2*), aEPEC (*eae* without either *bfpA*, *stx1*, or *stx2*), STEC (*eae* with *stx1* and/or *stx2*, and without *bfpA*), EAEC (*aatA* and/or *aaiC*), ST-ETEC (*STh* and/or *STp*, with or without *LT*), LT-ETEC (*LT*, without either *STh* or *STp*), EIEC/*Shigella* (*ipaH*), *Salmonella* (*ttr*), *C*. *jejuni/coli* (*cadF*), *V*. *cholerae* (*hlyA*). Based on our methods, enteropathogen positivity reflects the presence/absence of each enteropathogen in stool but does not measure absolute quantity.

### Statistical analysis

Data cleaning and statistical analysis were conducted using Stata SE version 14.2 (StataCorp, College Station, TX, USA). Statistically significant associations are reported at the p < 0.05 level.

Descriptive statistics were calculated for child- and household-level characteristics and the child’s health status, including micronutrient deficiency, inflammation, and indications of illness. Correlations between Hb, log-transformed SF and sTfR, and log-transformed CRP and AGP concentrations were examined to assess associations between the outcomes of interest. We assessed the overall prevalence of each pathogen in each child’s stool and the total number of pathogens detected per child. To determine whether children had symptomatic infections, we used logistic regression to model the associations between enteropathogen presence and diarrhea and other morbidity symptoms in the past seven days, adjusting for child age and sex.

Unadjusted and adjusted logistic regression were used to model the associations between the presence/absence of each enteropathogen and elevated inflammation, iron deficiency, and anemia. Anemia was defined as Hb < 110 g/L, per the WHO recommendations [45]. Adjusted models included the child’s sex and age in months, which were chosen *a priori* as potential confounders. Malaria parasitemia and breastfeeding status were considered for inclusion as confounders, but were not used in the final models since the inclusion of these variables did not meaningfully change effect estimates and because breastfeeding was strongly correlated with child age. Socioeconomic variables were not formally analyzed in this study of enteropathogen impacts, though such factors likely affect exposure risk. This approach is consistent with other similar studies that have investigated associations between infections and biological parameters, such as iron status [29] or EED biomarkers [46]. While sample selection was stratified based on household chicken ownership, owning chickens was not associated with enteropathogen detection in children [35]. As a sensitivity analysis, we used adjusted linear regression to model associations between enteropathogen detection and the outcomes of interest as continuous variables (i.e., CRP, AGP, SF, sTfR, and Hb concentrations).

Additional analyses were run to examine effects by relative enteropathogen load (high or low) and by pathogen groupings. High and low pathogen detection were defined as below the median Ct (high) and above the median Ct (low) for each target gene. Adjusted logistic regression models were used to predict inflammation, iron deficiency, and anemia by “high” or “low” relative target gene detection, in reference to no detection. We additionally assessed pathogen detection using three groupings: enteroinvasive bacteria (EIEC/*Shigella*, *C*. *jejuni/coli*, or *Salmonella*) and potentially enteroinvasive bacteria (EAEC or STEC), non-enteroinvasive, surface adherent bacteria (tEPEC, aEPEC, ST-ETEC, LT-ETEC), or co-detection of both enteroinvasive and non-enteroinvasive bacteria. Logistic regression was also used to evaluate the outcomes comparing detection of enteroinvasive pathogens and co-detection of enteroinvasive and non-enteroinvasive pathogens relative to only non-enteroinvasive pathogens, adjusting for child age and sex.

### Ethical considerations

This study was approved by the University of Michigan Health Sciences and Behavioral Sciences Institutional Review Board (protocol no. HUM00145171) and the Noguchi Memorial Institute for Medical Research Institutional Review Board (protocol no. 098/17-18). Before any data were collected, the survey team met with community leaders to explain the project and obtain their general approval. At each household, after explaining the study procedures, risks, and benefits, written informed consent was provided by the index child’s caregiver for their child’s participation in the study and their participation in the interview with a signature or thumbprint. Households were given a small, non-monetary gift as compensation for their participation. Children with malaria parasites or anemia were advised to visit their nearest health clinic to confirm the test result and receive professional care.

### Inclusivity in global research

Additional information regarding the ethical, cultural, and scientific considerations specific to inclusivity in global research is included in the **S1 Checklist**.

## Results

Of the 265 children’s stool samples that were analyzed for enteropathogens by qPCR, we excluded one sample with missing data on age and two samples lacking biomarker data, for a final analytic sample of 262 children (**Fig 1**). On average, children were 28 months old, with 47% of children between 6–23 months old and 53% between 24–59 months old (**Table 1**). Most households had access to an improved drinking water source, but access to adequate sanitation facilities varied, with almost one-third of households practicing open defecation, and less than 3% with a handwashing facility. Overall, 45.8% of children were anemic, of whom 46.7% had mild anemia, 50.0% had moderate anemia, and 3.3% had severe anemia (**Table 2**). One quarter of children had iron deficiency, as defined by low SF concentrations. Sixteen percent of children had elevated CRP concentrations while 37.8% had elevated AGP concentrations. No children had a detectable helminth infection, while 8.4% were positive for malaria parasitemia.

**Table 1:** Study sample characteristics of children aged 6–59 months old from Greater Accra Region, Ghana, October-November 2018 (n = 262).

Characteristics	Values
*Child characteristics*	
Sex (female); n (%)	127 (48.5)
Age (months); mean (SD)	27.7 (14.0)
6–11 months; n (%)	30 (11.5)
12–23 months; n (%)	94 (35.9)
24–35 months; n (%)	64 (24.4)
36–47 months; n (%)	46 (17.6)
48–59 months; n (%)	28 (10.7)
Currently breastfeeding; n (%)	82 (31.3)
*Household characteristics*	
Number of children under 5 years; mean (SD)	1.3 (0.6)
Head of household sex (female); n (%)	55 (21.0)
Maternal education; n (%)^1^	
None, nursery, or primary	120 (46.3)
Junior or higher	139 (53.7)
Improved drinking water source; n (%)^2^	255 (97.3)
Sanitation facility; n (%)	
Flush or pour-flush	28 (10.7)
Pit latrine	154 (58.8)
No facility/open defecation	80 (30.5)
Presence of handwashing facility; n (%)	7 (2.7)
Ownership of any livestock; n (%)	162 (61.8)
District; n (%)	
Ga East	91 (34.7)
Shai Osudoku	171 (65.3)

^1^n = 259

^2^Includes piped water, public pipe/standpipe, tube well or borehole, protected dug well, protected spring, bottled water/sachet water, cart with tank, and rain water.

**Table 2:** Micronutrient status, inflammation, and illness in Ghanaian children aged 6–59 months old (n = 262).

Indicator	Value
*Micronutrient status biomarkers* ^*1*^	
Hb (g/L); mean (SD)	109 (15)
Anemia; n (%)	
None (Hb ≥ 110 g/L)	142 (54.2)
Mild (Hb 100–109 g/L)	56 (21.4)
Moderate (Hb 70–99 g/L)	60 (22.9)
Severe (Hb < 70 g/L)	4 (1.5)
Iron deficiency anemia; n (%)^2^	47 (17.9)
SF (μg/L); median (IQR)	22.89 (12.19, 42.91)
Iron deficiency (SF < 12 μg/L); n (%)	64 (24.4)
sTfR (mg/L); median (IQR)	7.99 (6.60, 10.43)
Iron deficiency (sTfR > 8.3 mg/L); n (%)	116 (44.3)
*Inflammation biomarkers*	
CRP (mg/L); median (IQR)	0.52 (0.16, 2.24)
Inflammation (CRP > 5 mg/L); n (%)	43 (16.4)
AGP (g/L); median (IQR)	0.83 (0.58, 1.29)
Inflammation (AGP > 1 g/L); n (%)	99 (37.8)
*Infection and illness symptoms*	
Malaria; n (%)	22 (8.4)
Helminthiasis; n (%)^3^	0 (0.0)
Fever in past 7d; n (%)^4^	66 (25.2)
Diarrhea in past 7d; n (%)	17 (6.5)
Cough/cold in past 7d; n (%)	65 (24.8)
Nausea in past 7d; n (%)	15 (5.7)
Vomiting in past 7d; n (%)	16 (6.1)

^1^SF and sTfR are inflammation-adjusted values. Unadjusted SF values: 30.79μg/L (15.65, 56.09). Unadjusted sTfR values: 8.44mg/L (6.88, 11.15).

^2^Iron deficiency anemia is defined as both anemia and iron deficiency (SF < 12 μg/L).

^3^N = 257

^4^13.6% of fever associated with malaria.

Abbreviations: AGP, α-1-acid glycoprotein; CRP, C-reactive protein; Hb, hemoglobin; SF, serum ferritin; sTfR, serum transferrin receptor.

Biomarkers of iron status and inflammation were significantly correlated with hemoglobin concentration in the expected directions (**S2 Table**). The correlations between the iron biomarkers, SF and sTfR, and between the inflammatory biomarkers, CRP and AGP, were positive and strong (p < 0.0001). SF showed moderate, positive correlations with CRP and AGP (p < 0.001) before adjusting for inflammation, but not after, indicating that the BRINDA regression correction approach corrected artificially-elevated SF concentrations. sTfR was weakly associated with AGP before adjusting for inflammation (p = 0.026), but not CRP, and was not correlated with either inflammatory biomarker after adjusting for inflammation.

### Enteropathogen detection in children

**Fig 2:**
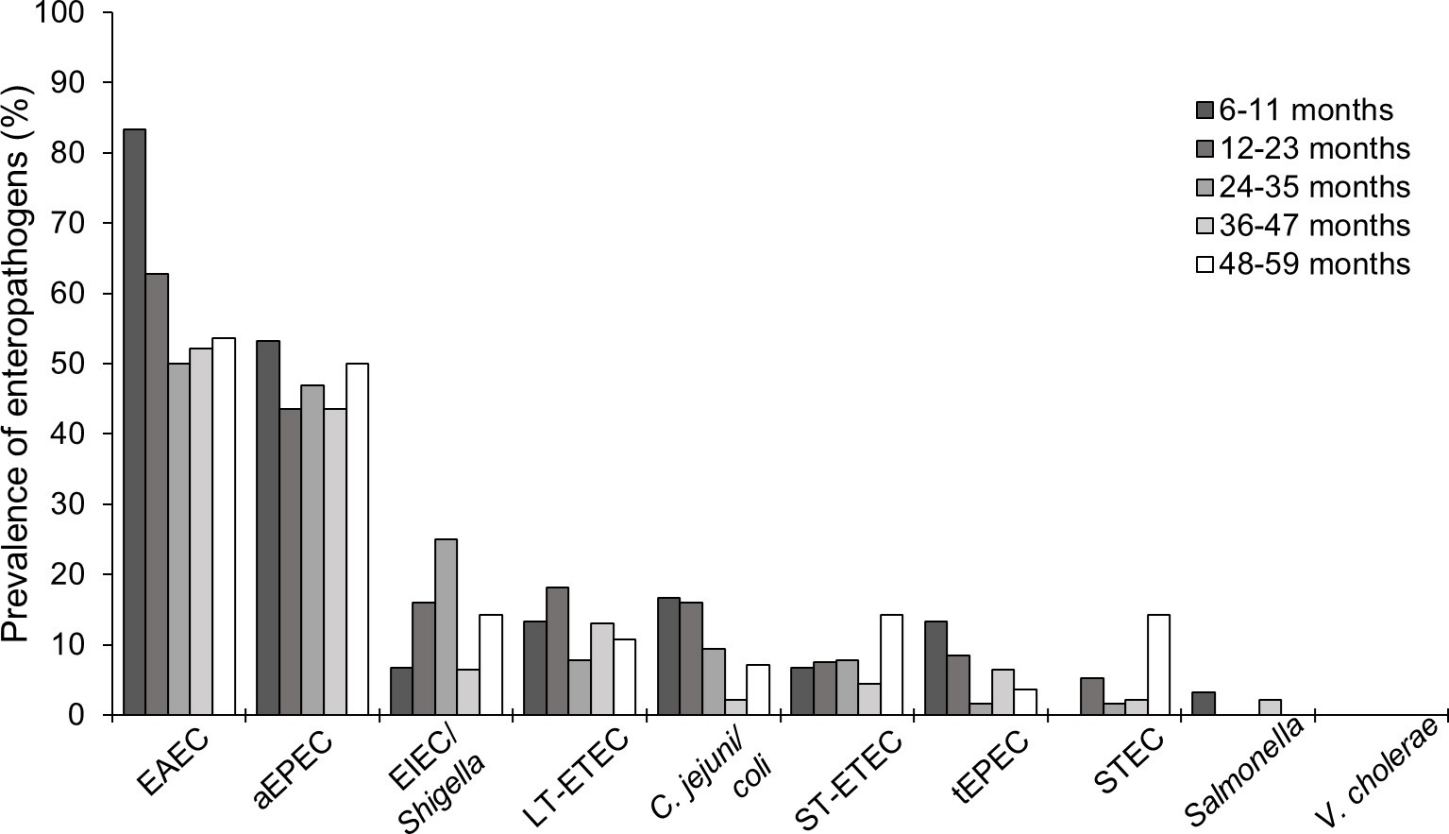
Prevalence of enteropathogens detected in children’s stool by age (months) in the Greater Accra Region, Ghana, October-November 2018 (n = 262). Age categories: 6–11 months (n = 30), 12–23 months (n = 94), 24–35 months (n = 64), 36–47 months (n = 46), 48–59 months (n = 28). Abbreviations: aEPEC, atypical enteropathogenic *Escherichia coli (E*. *coli)*; C. jejuni/coli, *Campylobacter jejuni* or *Campylobacter coli*; EAEC, enteroaggregative *E*. *coli*; EIEC, enteroinvasive *E*. *coli*; LT-ETEC, heat-labile enterotoxin-producing *E*. *coli*; STEC, Shiga toxin-producing *E*. *coli;* ST-ETEC, heat-stable enterotoxin-producing *E*. *coli;* tEPEC, typical enteropathogenic *E*. *coli*.

Enteropathogens were detected in 87.0% of children’s stool (**Table 3**). One-third of children were positive for one pathogen, one-third for two pathogens, and three or four pathogens were detected in one-fifth of children. EAEC and aEPEC were most common, detected in 59.2% and 46.2% of children’s stool, respectively. Among the other pathogens assessed, 15.3% of stool samples were positive for EIEC/*Shigella*, 13.4% for LT-ETEC, 11.1% for *C*. *jejuni/coli*, 7.6% for ST-ETEC, 6.5% for tEPEC, and 4.2% for STEC. *Salmonella* was rare (detected in only two children) and *V*. *cholerae* was not detected. Enteropathogens were found across all sampled age ranges (**Fig 2**). EAEC was more prevalent among children 6–12 months old than older children, while STEC was more prevalent among children 4–5 years old than the younger age ranges.

**Table 3:** Prevalence of enteropathogens detected in stool of Ghanaian children aged 6–59 months old (n = 262).

Pathogen	N (%)
EAEC	155 (59.2)
aEPEC	121 (46.2)
EIEC/*Shigella*	40 (15.3)
LT-ETEC	35 (13.4)
*C*. *jejuni/coli*	29 (11.1)
ST-ETEC	20 (7.6)
tEPEC	17 (6.5)
STEC	11 (4.2)
*Salmonella*	2 (0.8)
*V*. *cholerae*	0 (0.0)
Any pathogen	
Yes	228 (87.0)
No	34 (13.0)
Number of detected pathogens per sample^1^	
0	34 (13.0)
1	92 (35.1)
2	82 (31.3)
3	42 (16.0)
4	12 (4.6)
≥5	0 (0.0)

^1^Sum ranges from 0 to 10.

Abbreviations: aEPEC, atypical enteropathogenic *Escherichia coli (E*. *coli)*; *C*. *jejuni/coli*, *Campylobacter jejuni* or *Campylobacter coli*; EAEC, enteroaggregative *E*. *coli*; EIEC, enteroinvasive *E*. *coli*; LT-ETEC, heat-labile enterotoxin-producing *E*. *coli*; STEC, Shiga toxin-producing *E*. *coli;* ST-ETEC, heat-stable enterotoxin-producing *E*. *coli;* tEPEC, typical enteropathogenic *E*. *coli*.

### Associations between enteropathogen detection and indicators of morbidity and inflammation

Reported diarrhea prevalence in the preceding seven days was 6.5%. Of the 17 children with diarrhea, caregivers indicated five (29%) had watery diarrhea and none had bloody diarrhea. In bivariate analysis, EAEC was marginally associated with diarrhea [Odds Ratio (OR) (95% CI): 3.44 (0.96, 12.29); p = 0.057]. After adjusting for child age and sex, only STEC was associated with diarrhea [OR (95% CI): 5.78 (1.02, 32.64); p = 0.047] (**S3 Table**). No other morbidity symptoms (fever, cough or cold, nausea, or vomiting) were associated with enteropathogen detection.

**Fig 3:**
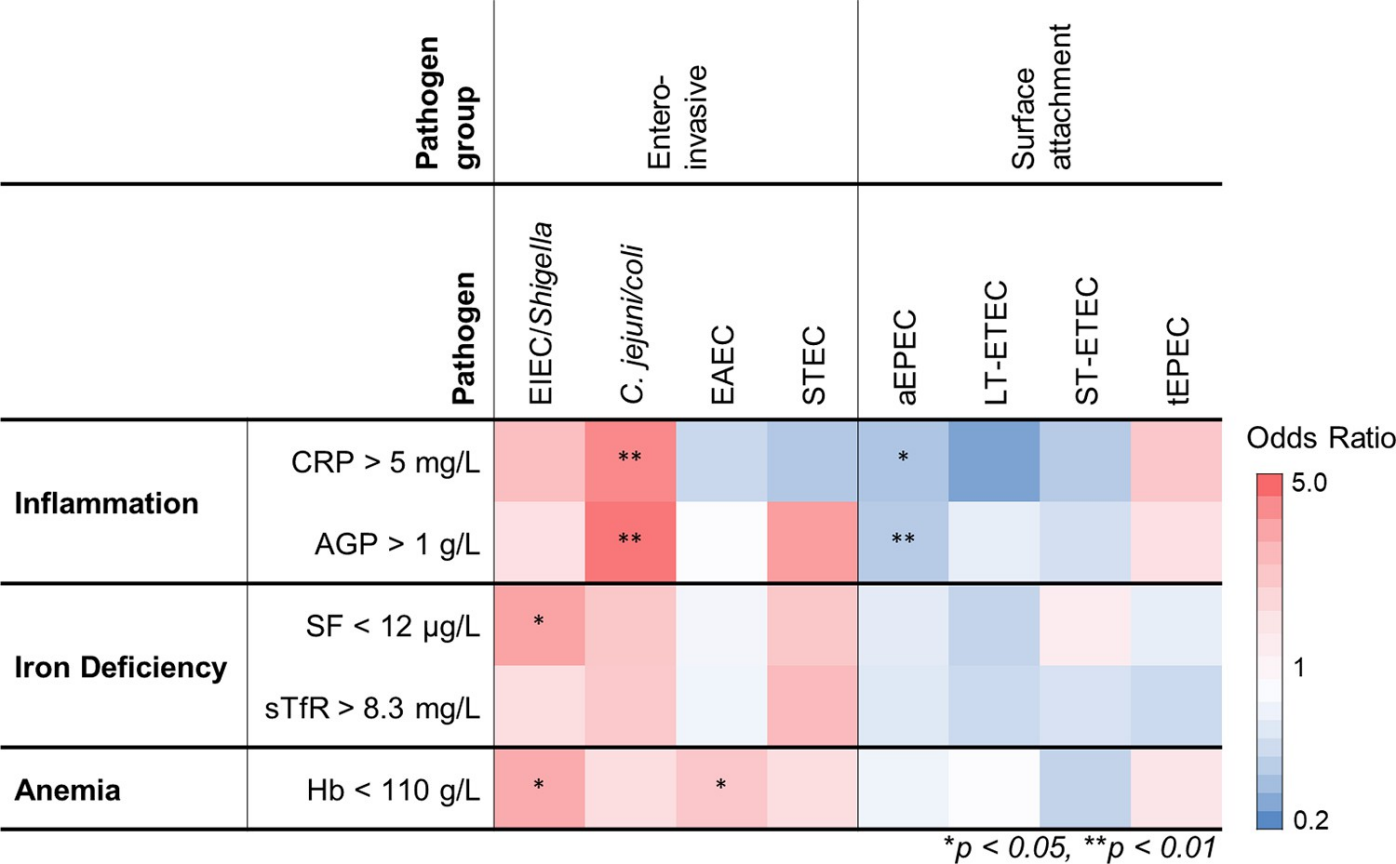
Associations between enteropathogen detection in children’s stool and systemic inflammation, iron deficiency, and anemia among children aged 6–59 months old in Greater Accra, Ghana (n = 262). Higher odds of the outcome are represented in red and lower odds of the outcome are represented in blue, with darker shades of the colors representing stronger associations. Odds ratios and significance values are derived from adjusted logistic regression models controlling for child age and sex (S4 Table). Abbreviations: aEPEC, atypical enteropathogenic *Escherichia coli (E*. *coli)*; AGP, α-1-acid glycoprotein; *C*. *jejuni/coli*, *Campylobacter jejuni* or *Campylobacter coli*; CRP, C-reactive protein; EAEC, enteroaggregative *E*. *coli*; EIEC, enteroinvasive *E*. *coli*; Hb, hemoglobin; LT-ETEC, heat-labile enterotoxin-producing *E*. *coli*; SF, serum ferritin; STEC, Shiga toxin-producing *E*. *coli;* ST-ETEC, heat-stable enterotoxin-producing *E*. *coli;* sTfR, serum transferrin receptor; tEPEC, typical enteropathogenic *E*. *coli*.

*C*. *jejuni/coli* detection was associated with 3.49 times higher odds of elevated CRP concentrations (95% CI: 1.45, 8.41) and 4.27 times higher odds of elevated AGP concentrations (95% CI: 1.85, 9.84), controlling for child age and sex (**Fig 3 and S4 Table**). Associations between *C*. *jejuni/coli* and elevated inflammation were found at both high and low relative detection of the *cadF* gene (**S5 Table**). An association between EIEC/*Shigella* and inflammation (CRP) was observed only at high relative quantity of the *ipaH* gene [OR (95% CI): 3.24 (1.18, 8.89)]. In grouped analysis, children with detection of at least one enteroinvasive bacterium (*C*. *jejuni/coli*, EAEC, EIEC/*Shigella*, STEC and/or *Salmonella*) had higher odds of elevated AGP in comparison to children with detection of non-enteroinvasive bacterium species (**Table 4**). Detection of aEPEC in stool was associated with lower odds of elevated CRP and AGP (S4 Table).

**Table 4:** Adjusted odds of elevated systemic inflammation, iron deficiency, and anemia comparing enteroinvasive and enteroinvasive/non-enteroinvasive pathogen co-detection in reference to non-enteroinvasive pathogen detection among Ghanaian children 6–59 months old^1^.

Pathogen group^2^	N	Inflammation		Iron Deficiency		Anemia
		CRP > 5 mg/L	AGP > 1 g/L	SF < 12 μg/L	sTfR > 8.3 mg/L	Hb < 110 g/L
Non-enteroinvasive only	49	Ref	Ref	Ref	Ref	Ref
Enteroinvasive and non-enteroinvasive co-detection	113	1.75 (0.54, 5.68)	1.57 (0.73, 3.40)	0.96 (0.41, 2.25)	1.03 (0.51, 2.10)	2.10* (1.00, 4.40)
Enteroinvasive only	66	3.02 (0.91, 10.02)	3.76** (1.65, 8.56)	2.05 (0.84, 4.97)	2.22* (1.03, 4.80)	2.85* (1.27, 6.38)

^1^Values are Odds Ratio (95% Confidence Interval) using adjusted logistic regression models, controlling for child sex and age in months

*p<0.05

**p<0.*01*. SF and sTfR cut-offs use inflammation-adjusted values. Sample size: n = 228; Abbreviations: Hb, hemoglobin; SF, serum ferritin; sTfR, serum transferrin receptor; CRP, C-reactive protein; AGP, α-1-acid glycoprotein.

^2^The enteroinvasive only pathogen group includes children with detection of one or more enteropathogens that invade (*Campylobacter jejuni/coli*, enteroinvasive *E*. *coli/Shigella*, *Salmonella*) or potentially invade [enteroaggregative *E*. *coli* (EAEC), Shiga toxin-producing *E*. *coli* (STEC)] intestinal epithelial cells. The non-enteroinvasive pathogen only group includes children with detection of one or more enteropathogens that are not known to invade intestinal epithelial cells [enteropathogenic *E*. *coli* (EPEC), enterotoxin-producing *E*. *coli* (ETEC)]. The enteroinvasive and non-enteroinvasive pathogen group includes children with detection of both invasive and non-invasive enteropathogens in their stool.

### Associations between enteropathogen detection and iron status and anemia

An association between enteropathogen detection and iron deficiency was observed among children infected with EIEC/*Shigella* (Fig 3). Children with EIEC/*Shigella* had 2.55 times higher odds of low SF concentrations (95% CI: 1.23, 5.29). Neither detection of EIEC/*Shigella* nor other individual enteropathogens was associated with elevated sTfR concentrations. However, detection of one or more enteroinvasive pathogens as a group was associated with higher odds of elevated sTfR concentrations compared to detection of non-enteroinvasive pathogens (Table 4).

EAEC (OR: 1.69, 95% CI: 1.01, 2.84) and EIEC/*Shigella* (OR: 2.34, 95% CI: 1.15, 4.76) were associated with higher odds of anemia, adjusting for child age and sex (Fig 3). In sensitivity analyses using linear regression, detection of EIEC/*Shigella* in stool was associated with lower Hb concentrations (β = ˗5, 95% CI: ˗1, ˗0.3) (**S6 Table**). Furthermore, children with one or more enteroinvasive bacteria had higher odds of anemia compared to children with only non-enteroinvasive bacteria (Table 4).

## Discussion

This cross-sectional study investigated the role of enteropathogens in the etiology of anemia among 6-59-month-old children in semi-rural communities of Greater Accra, Ghana. Anemia affected almost half of children, though less than one-fifth of children had iron deficiency anemia. Almost forty percent of children had elevated CRP or AGP concentrations, indicative of inflammatory responses to infection. These results alongside the finding that CRP and AGP were strongly correlated with Hb concentrations suggest that anemia in this sample is in part attributable to infectious factors, in agreement with recent studies of anemia in Ghanaian children [10, 11]. As has been found in other studies investigating the burden of enteropathogens among children in LMICs, most children (87.0%) in this study had at least one bacterial enteropathogen detected in their stool, with EAEC and aEPEC being the most prevalent [20, 47]. Enteropathogens presented as predominantly subclinical, with only one in every 15 children experiencing diarrhea. Despite few apparent clinical symptoms, the presence of *Campylobacter* in stool was strongly associated with systemic inflammation. Interestingly, EAEC detection was associated with anemia, but not with iron deficiency. However, EIEC/*Shigella* was associated with both iron deficiency and anemia, and children positive for EIEC/*Shigella* exhibited 5 g/L lower Hb concentrations on average than children without EIEC/*Shigella*.

Several recent studies have linked indicators of enteropathogen exposure, such as poor water and sanitation conditions [48, 49] and biomarkers of EED [28, 29, 31], to lower hemoglobin concentrations and anemia in young children, prompting the hypothesis that enteropathogen infections are directly linked to anemia [14]. Analysis of the MAL-ED (Etiology, Risk Factors, and Interactions of Enteric Infections and Malnutrition and the Consequences for Child Health) multi-site birth cohort study indeed demonstrated that the mean number of different parasitic, viral, and bacterial enteropathogens detected in both diarrheal and non-diarrheal stools across children’s first 24 months of life was associated with lower hemoglobin concentrations [23]. The analysis by the MAL-ED Network investigators is one of the few studies to our knowledge that has directly linked enteropathogen infection to anemia. Additional evidence comes from iron fortification trials. An unfavorable ratio of pathogenic enterobacteria to commensal lactobacilli and bifidobacteria was observed at baseline in anemic school-age children in a fortification trial in Côte d’Ivoire [32]. Similarly, an iron fortification trial in Kenya observed that anemic 6-month-old infants harbored a higher abundance of pathogenic bacteria in their gut microbiome compared to non-anemic infants [33]. Iron supplementation further increased gut colonization by pathogenic bacteria, including *Escherichia*/*Shigella* and *E*. *coli* pathotypes, and gut inflammation among anemic African children [32] and infants [33, 50]. Our study adds to this growing body of literature and presents novel findings which suggest that infections with specific bacteria, rather than enteropathogen exposure broadly, may contribute to the development of anemia.

The pathogens that were most associated with systemic inflammation, iron deficiency, and anemia in this study were enteroinvasive bacteria that typically penetrate intestinal epithelial cells and produce tissue destruction and enteric inflammation [51–54]. Notably, subclinical detection of these enteropathogens (EAEC, *Campylobacter*, and EIEC/*Shigella*) are also the most deleterious for linear growth faltering [17, 20]. Systemic inflammation, more so than gut inflammation, appears to be a key mediator in the associations between intestinal pathogen exposure, particularly enteroinvasive bacterial pathogens, and child growth [17, 55]. This mechanism is likely also responsible for iron dysregulation and anemia among children, as systemic inflammation inhibits absorption of iron and induces iron sequestration via upregulation of hepcidin. Indeed, systemic inflammation has been shown to predict hepcidin concentrations in African infants [13, 56]. Prentice et al. (2019) found that almost half of Gambian children 6 to 27 months old were physiologically blocking iron absorption, and that CRP concentrations, even at low levels, were correlated with hepcidin concentrations [13]. It is thus plausible that systemic inflammation associated with enteroinvasive pathogens may produce anemia via elevated hepcidin. Future studies are needed to confirm this as we did not measure hepcidin concentrations in children due to financial constraints.

Given findings from prior studies that gut permeability and gut inflammation are associated with iron status and anemia in children, EED may be an additional pathway by which enteropathogen infections can lead to anemia [28, 29, 31]. Though causal links between EED and anemia have not been elucidated [12], gut dysfunction and gut inflammation, combined with a systemic inflammatory response to acute infection, likely mediate associations between enteropathogens and anemia, though we did not measure EED biomarkers directly. Our findings suggest that these mechanisms may differ by enteropathogen.

Among the enteroinvasive pathogens, only EIEC/*Shigella* detection was associated with both systemic inflammation and alterations in iron status and anemia. EIEC/*Shigella* detection at higher relative quantities was associated with elevated inflammation and overall detection of this pathogen was associated with higher odds of low ferritin concentrations and anemia. Given the short duration of *Shigella* incubation (1–4 days) and infection (2–3 days) [57], it is plausible that the association observed between detection of the *Shigella ipaH* gene and CRP at higher relative quantities, but not at lower relative quantities, reflects an acute systemic inflammatory response. Subclinical *Shigella* infection has been associated with intestinal and systemic inflammation [46], thus *Shigella* may reduce iron availability necessary for hematopoiesis via reduced micronutrient absorption and systemic inflammation. While *Shigella* infection can cause dysentery, that no children in our study were reported to have had bloody stool in the previous seven days suggests this does not explain the association with anemia.

Detection of EAEC in stool was associated with anemia, but not with iron deficiency nor with elevated systemic inflammation. EAEC is one of the most pervasive and persistent enteric infections detected in young children in LMICs [16, 19, 20]. Analysis from the MAL-ED birth cohort found that frequent EAEC detection was associated with long-term growth faltering, but not with short-term growth velocity in weight or length [19]. In parallel, it is possible that repeated exposure to EAEC contributes to anemia in children. As our study did not evaluate repeat infections, further research is needed to investigate whether frequent EAEC detection in early life contributes to anemia. As in our study, EAEC detection among the MAL-ED birth cohort was not associated with elevated AGP [19], though subclinical EAEC detection has been related to intestinal inflammation [19, 58], with inflammatory effects possibly dependent upon co-infections [59].

A strong association between *Campylobacter jejuni/coli* detection and systemic inflammation has been shown previously for AGP [18] and is consistent with *Campylobacter’s* induction of a robust immune response following epithelial invasion [52, 54]. However, neither an association with anemia nor with iron deficiency was observed, suggesting that the inflammatory response induced by *Campylobacter* may not alter iron homeostasis. Interestingly, aEPEC was associated with lower odds of systemic inflammation in our study. Such reduced inflammatory response may reflect the pathogenic mechanism by which EPEC persists in the intestine–evading the mucosal immune system by inhibiting cell cytokine secretion and NF-ĸB signaling (which is upstream of CRP and AGP production) [60]. Furthermore, co-detection of non-enteroinvasive bacteria (EPEC or ETEC) and enteroinvasive bacteria was not associated with a higher inflammatory response compared to non-enteroinvasive detection alone. Additional epidemiologic studies are needed to confirm these findings and to investigate whether co-infections of EPEC and enteroinvasive pathogens interact to alter systemic inflammation.

This study examined associations between enteropathogen detection and anemia in children using both molecular diagnostics to identify several individual bacterial pathogens and multiple biomarkers for systemic inflammation and iron status to better understand potential mechanisms underlying the observed associations. However, there were several limitations. First, we could not ascertain temporal relationships between enteropathogen detection and the outcomes given the cross-sectional study design. Since inflammation may occur days after an infection, with iron deficiency and anemia taking several weeks to develop, concurrent collection of stool and blood samples precluded longitudinal assessment of relationships. Thus, observed associations with anemia can reflect either prior infections or persistent infections, which we did not measure. Furthermore, the high sensitivity of molecular diagnostics means that pathogen detection can represent clinically-relevant infections as well as low-level pathogen exposure and carriage without colonization [43]. Identifying clinically-relevant cut-offs for enteropathogen detection, particularly of asymptomatic infections, remains a work in-progress. Second, the study sample size was smaller than that of many multi-site studies, which may have lowered our power to detect associations, especially among pathogens with a low prevalence, even though our sample size was within the range of per-site sample sizes in the MAL-ED birth cohort [31]. Third, since data were collected in only one region of Ghana and stratified by chicken ownership, enteropathogen prevalence estimates cannot be generalized to all children in this or other regions of Ghana. Finally, children’s nutrient intake may influence their susceptibility to enteropathogenic infections via differences in gut function [31], which we did not measure in our study. Thus, further investigations into the role of diet in modifying associations between infection and anemia are warranted.

## Conclusion

Our findings suggest that subclinical presence of specific invasive enteropathogenic bacteria, particularly EAEC and EIEC/*Shigella*, may contribute to anemia in young children. Prior literature has found that poor water and sanitation are associated with anemia among young children living in LMICs [48, 49], with the hypothesis that exposure to enteropathogens underlies this relationship [14]. By demonstrating an association between certain enteropathogens and anemia, our results lend direct evidence to support this hypothesis. Addressing subclinical enteric infections through improved water, sanitation, and hygiene infrastructure and other approaches such as vaccines should help reduce the anemia burden among young children in Ghana and other similar settings. Additional studies are needed to confirm our findings and assess the relative contribution of enteropathogens to children’s anemia burden, as compared to other infectious diseases and micronutrient deficiencies. Furthermore, research involving longitudinal assessment of individual enteropathogens and iron deficiency and anemia, alongside EED markers, hepcidin, and systemic inflammatory biomarkers, would improve our understanding of the mechanisms linking bacterial enteropathogens and anemia among young children.

## Supporting information

S1 Checklist Inclusivity in global research. (DOCX)

S2 Checklist STROBE statement. (DOCX)

S1 Data Dataset. (CSV)

S1 Table Primer and probe sequences for qPCR detection of pathogen gene targets and assay performance characteristics. (DOCX)

S2 Table Correlation matrix between hemoglobin concentration, iron status biomarkers (SF and sTfR), and inflammatory biomarkers (CRP and AGP) among 262 children aged 6–59 months old in Greater Accra, Ghana. (DOCX)

S3 Table Adjusted associations between enteropathogen detection and symptoms of illness among children aged 6–59 months old in Greater Accra, Ghana. (DOCX)

S4 Table Adjusted associations between enteropathogen detection and elevated systemic inflammation, iron deficiency, and anemia among 262 Ghanaian children aged 6–59 months old. (DOCX)

S5 Table Adjusted associations of high and low relative gene target quantity and inflammation (CRP and AGP), iron deficiency (SF and sTfR), and anemia (Hb) among children aged 6–59 months old in Greater Accra, Ghana. (DOCX)

S6 Table Adjusted associations between enteropathogen detection and concentrations of inflammatory biomarkers (CRP and AGP), iron status biomarkers (SF and sTfR), and hemoglobin (Hb) among children aged 6–59 months old in Greater Accra, Ghana. (DOCX)
