# Supplementary material for: Associations of bacterial enteropathogens with systemic inflammation, iron deficiency, and anemia in preschool-age children in southern Ghana
Source: PLoS One. 2022 Jul 8;17(7):e0271099. doi: 10.1371/journal.pone.0271099 (PMC9269377; doi:10.1371/journal.pone.0271099)
Supplement: S2 Checklist — (DOCX) [file pone.0271099.s002.docx]

S2 Checklist. STROBE Statement

|  | Item No. | Recommendation | Page  No. | Relevant text from manuscript |
| --- | --- | --- | --- | --- |
| **Title and abstract** | 1 | (*a*) Indicate the study’s design with a commonly used term in the title or the abstract | 2 | Serum samples were analyzed from a cross-sectional sample of 262 children |
|  |  | (*b*) Provide in the abstract an informative and balanced summary of what was done and what was found | 2-3 | Anemia remains a pervasive public health problem among preschool-age children in Ghana. Recent analyses have found that anemia in Ghanaian children, particularly in Southern regions, is largely attributable to infectious causes, rather than nutritional factors. Infections with enteropathogens can reduce iron absorption and increase systemic inflammation, but few studies have examined direct links between enteropathogens and anemia. This study investigated associations between detection of individual bacterial enteropathogens and systemic inflammation, iron deficiency, and anemia among 6- to 59-month-old children in Greater Accra, Ghana. Serum samples were analyzed from a cross-sectional sample of 262 children for concentrations of hemoglobin (Hb), biomarkers of systemic inflammation [C-reactive protein (CRP) and α-1-acid glycoprotein (AGP)], and biomarkers of iron status [serum ferritin (SF) and serum transferrin receptor (sTfR)]. Stool samples were analyzed for ten bacterial enteropathogens using qPCR. We estimated associations between presence of each enteropathogen and elevated systemic inflammation (CRP > 5 mg/L and AGP > 1 g/L), iron deficiency (SF < 12 µg/L and sTfR > 8.3 mg/L) and anemia (Hb < 110 g/L). Enteropathogens were detected in 87% of children’s stool despite a low prevalence of diarrhea (6.5%) . Almost half (46%) of children had anemia while one-quarter (24%) had iron deficiency (low SF). Despite finding no associations with illness symptoms, Campylobacter jejuni/coli detection was strongly associated with elevated CRP [Odds Ratio (95% CI): 3.49 (1.45, 8.41)] and elevated AGP [4.27 (1.85, 9.84)]. Of the pathogens examined, only enteroinvasive Escherichia coli/Shigella spp. (EIEC/Shigella) was associated with iron deficiency, and enteroaggregative Escherichia coli (EAEC) [1.69 (1.01, 2.84)] and EIEC/Shigella [2.34 (1.15, 4.76)] were associated with anemia. These results suggest that certain enteroinvasive pathogenic bacteria may contribute to child anemia. Reducing exposure to enteropathogens through improved water, sanitation, and hygiene practices may help reduce the burden of anemia in young Ghanaian children. |
| Introduction |  |  |  |  |
| Background/rationale | 2 | Explain the scientific background and rationale for the investigation being reported | 4-5 | Studies have linked subclinical enteric pathogen carriage to linear growth faltering and cognitive development delays, which are posited to occur, in part, through a condition of gastrointestinal inflammation and permeability known as environmental enteric dysfunction (EED) [17–23]. There are two plausible mechanisms by which enteropathogen infections may also lead to iron deficiency and anemia.  Observational studies have identified positive associations between EED biomarkers and anemia, supporting a link between enteropathogen exposure and anemia [28–31]. A few studies that have measured enteropathogens provide further evidence for this association. A longitudinal birth cohort study conducted in eight LMICs found that higher rates of enteropathogen detection in stool over children’s first two years of life was associated with lower hemoglobin concentrations [23]. Analyses of the baseline microbiome of anemic school-age children in Côte d’Ivoire and anemic 6-month-old infants in Kenya enrolled in iron fortification trials revealed an adverse abundance of pathogenic enterobacteria and lower ratio of commensal bacteria when compared to non-anemic children [32,33]. Among the school-age children, however, no correlations between the number of fecal enterobacteria and baseline iron biomarker concentrations were observed [32]. Building on this body of literature, our study is the first to examine pathogen-specific associations with iron status and anemia among African preschool-age children. |
| Objectives | 3 | State specific objectives, including any prespecified hypotheses | 5-6 | In this study, we assessed the associations between detection of specific bacterial enteropathogens and elevated inflammation, iron deficiency, and anemia in children 6-59 months old in Greater Accra, Ghana. We hypothesized that detection of enteropathogens would be associated with higher odds of systemic inflammation, iron deficiency, and anemia. We did not have a priori hypotheses about the independent effect of bacterial pathogens on iron status and anemia, and thus analyzed each pathogen with all the outcomes of interest. |
| Methods |  |  |  |  |
| Study design | 4 | Present key elements of study design early in the paper | 6 | This cross-sectional study analyzed a subsample of 6-59 month old children from a study conducted in the Greater Accra region, Ghana investigating livestock ownership and anemia [33]. |
| Setting | 5 | Describe the setting, locations, and relevant dates, including periods of recruitment, exposure, follow-up, and data collection | 6 | Overall, 484 households from 18 semi-rural communities in the Ga East and Shai Osudoku districts were sampled between October and November 2018. Communities ranged in population size from small (less than 750 residents) to large (over 2,000 residents). |
| Participants | 6 | (*a*) *Cohort study*—Give the eligibility criteria, and the sources and methods of selection of participants. Describe methods of follow-up  *Case-control study*—Give the eligibility criteria, and the sources and methods of case ascertainment and control selection. Give the rationale for the choice of cases and controls  *Cross-sectional study*—Give the eligibility criteria, and the sources and methods of selection of participants | 6 | From 13 to 40 households were sampled per community, depending on community size, each with at least one child aged 6-59 months old (the index child). In households with more than one child in the target age range, the youngest child was recruited to participate. To be eligible for inclusion, the index child’s primary caregiver, usually their mother, had to be at least 18 years old.  From the sample of 484 children, 430 (89%) had available stool samples while from 54 children, stool samples were unable to be obtained (Fig 1). Of those with a stool sample, a subsample of 265 children (62%) was selected for analysis as follows due to the financial constraints of conducting enteropathogen testing. Based on an associated study investigating whether chicken ownership and detection of pathogenic bacteria in chickens’ feces is associated with enteropathogen infection in children [34], this 265-child subsample comprised all 163 children from chicken-owning households and 102 out of 267 children (38%) from a random sample of non-chicken owning households. |
|  |  | (*b*) *Cohort study*—For matched studies, give matching criteria and number of exposed and unexposed  *Case-control study*—For matched studies, give matching criteria and the number of controls per case |  |  |
| Variables | 7 | Clearly define all outcomes, exposures, predictors, potential confounders, and effect modifiers. Give diagnostic criteria, if applicable | 8  10  11 | Biomarker cut-offs for iron deficiency (SF and sTfR) and inflammation (CRP and AGP) were defined per the BRINDA project cut-offs for preschool-age children: inflammation-adjusted SF < 12 µg/L, inflammation-adjusted sTfR > 8.3 mg/L, CRP > 5 mg/L, and AGP > 1 g/L [38].  Positivity for enteropathogens was defined by presence of gene targets as follows: tEPEC (*eae* with *bfpA*, and without *stx1* or *stx2*), aEPEC (*eae* without either *bfpA*, *stx1*, or *stx2*), STEC (*eae* with *stx1* and/or *stx2*, and without *bfpA*), EAEC (*aatA* and/or *aaiC*), ST-ETEC (*STh* and/or *STp*, with or without *LT*), LT-ETEC (*LT*, without either *STh* or *STp*), EIEC/*Shigella* (*ipaH*), *Salmonella* (*ttr*), *C. jejuni/coli* (*cadF*), *V. cholerae* (*hlyA*).  Unadjusted and adjusted logistic regression were used to model the associations between the presence/absence of each enteropathogen and elevated inflammation, iron deficiency, and anemia. Anemia was defined as Hb < 110 g/L, per the WHO recommendations [45]. Adjusted models included the child’s sex and age in months, which were chosen a priori as potential confounders. Malaria parasitemia and breastfeeding status were considered for inclusion as confounders, but were not used in the final models since the inclusion of these variables did not meaningfully change effect estimates and because breastfeeding was strongly correlated with child age. |
| Data sources/ measurement | 8* | For each variable of interest, give sources of data and details of methods of assessment (measurement). Describe comparability of assessment methods if there is more than one group | 7  7  7-8  8  9  9-10 | Trained enumerators conducted interviews with each index child’s primary caregiver, recording information using electronic tablets (Samsung Galaxy Tab A, Model Number SM-T285) with the Qualtrics survey platform (Qualtrics, Provo, UT, USA). Data were collected on the household’s sociodemographic characteristics and the index child’s age and health.  Child capillary blood samples were collected using a finger-prick [36]. After wiping away the first drop of blood, hemoglobin concentration (Hb) was measured using a HemoCue® Hb 201+ portable hemoglobinometer (HemoCue AB, Ängelholm, Sweden) and malaria parasitemia was measured using the SD Malaria Ag P.f (HRP2/pLDH) antigen rapid diagnostic test (RDT) (Standard Diagnostics Inc., Gyeonggi‐do, Republic of Korea).  Serum samples were analyzed for serum ferritin (SF), serum transferrin receptor (sTfR), C-reactive protein (CRP), and α-1-acid glycoprotein (AGP) using a sandwich enzyme-linked immunosorbent assay (ELISA) [37].  Stool samples were analyzed for the soil-transmitted helminths *Ascaris lumbricoides, Trichuris trichiura, Ancylostoma duodenale*, and *Necator americanus* using Kato Katz methods [39]. Following duplicate thick smear preparation of each stool sample, two laboratory technicians independently analyzed the slides by microscopy for helminth eggs.  Microbial nucleic acid was extracted from stool samples using the QIAamp® PowerFecal® DNA Kit (Qiagen, Hilden, Germany). Following brief thawing, ~250 mg of stool was weighed and placed in a bead beating tube under a sterile hood. All extraction steps were then followed according to the kit protocol. In the final extraction step, 100 µL of DNA was eluted into an Eppendorf™ DNA LoBind microcentrifuge tube (Eppendorf, Hamburg, Germany). Each batch of nucleic acid extractions included an extraction blank to control for laboratory contamination, which went through all the extraction steps but without any addition of stool sample. Total DNA concentration and purity were measured using a NanoDrop™ 2000 spectrophotometer (ThermoFisher Scientific, Waltham, MA, USA). DNA samples were transported to the University of Michigan (Ann Arbor, MI, USA) and stored at ˗80°C until further analysis.  Taqman probe-based quantitative polymerase chain reaction (qPCR) was used to analyze DNA samples for the following bacterial enteropathogens: *Campylobacter jejuni*/*Campylobacter coli* (*C. jejuni/coli*), enteroaggregative *Escherichia (E.) coli* (EAEC), atypical enteropathogenic *E. coli* (aEPEC), typical enteropathogenic *E. coli* (tEPEC), heat-stable enterotoxin-producing *E. coli* (ST-ETEC), heat-labile enterotoxin-producing *E. coli* (LT-ETEC), *Salmonella enterica*, Shiga toxin-producing *E. coli* (STEC), enteroinvasive *E. coli/Shigella* species (EIEC/*Shigella*), and *Vibrio cholerae* (*V. cholerae*). Primer and probe sequences to identify pathogen gene targets were derived from Liu et al. [40] and Taniuchi et al. [41] (**S1 Table**).  DNA samples and extraction blanks were run single-plex for each gene target on 384-well plates using the QuantStudio™ 5 System (Applied Biosystems™, Foster City, CA, USA). DNA samples were diluted 1:10 in ddH_2_O and run in triplicate or quadruplicate. Each plate also included a water control (ddH_2_O in place of DNA). Each amplification well contained 4.5 µL 1:10-diluted DNA sample, 5.0 µL TaqMan™ Fast Advanced Master Mix (Applied Biosystems™, Foster City, CA, USA), and 0.5 µL of primer-probe mixture at a final concentration of 500:250 nM primer:probe. Samples went through the following cycling conditions: 95°C for 10 minutes followed by 45 amplification cycles of 95°C for 15 s and 60°C for 1 min. For the *cadF* amplicon, annealing and extension were at 58°C for 1 min. |
| Bias | 9 | Describe any efforts to address potential sources of bias | 6 | Children included in the subsample were not significantly different in sex or age from those who were excluded. |
| Study size | 10 | Explain how the study size was arrived at | 6  13 | See #6  Of the 265 children’s stool samples that were analyzed for enteropathogens by qPCR, we excluded one sample with missing data on age and two samples lacking biomarker data, for a final analytic sample of 262 children (**Fig 1**). |
| Quantitative variables | 11 | Explain how quantitative variables were handled in the analyses. If applicable, describe which groupings were chosen and why | 8  10  11  12 | Biomarker cut-offs for iron deficiency (SF and sTfR) and inflammation (CRP and AGP) were defined per the BRINDA project cut-offs for preschool-age children: inflammation-adjusted SF < 12 µg/L, inflammation-adjusted sTfR > 8.3 mg/L, CRP > 5 mg/L, and AGP > 1 g/L [38].  A Ct cut-off of ≤ 35 was applied to define positive detection of a target gene.  Anemia was defined as Hb < 110 g/L, per the WHO recommendations [45].  Additional analyses were run to examine effects by relative enteropathogen load (high or low) and by pathogen groupings. High and low pathogen detection were defined as below the median Ct (high) and above the median Ct (low) for each target gene. |
| Statistical methods | 12 | (*a*) Describe all statistical methods, including those used to control for confounding | 11  12 | Descriptive statistics were calculated for child- and household-level characteristics and the child’s health status, including micronutrient deficiency, inflammation, and indications of illness. Correlations between Hb, log-transformed SF and sTfR, and log-transformed CRP and AGP concentrations were examined to assess associations between the outcomes of interest. We assessed the overall prevalence of each pathogen in each child’s stool and the total number of pathogens detected per child. To determine whether children had symptomatic infections, we used logistic regression to model the associations between enteropathogen presence and diarrhea and other morbidity symptoms in the past seven days, adjusting for child age and sex.  Unadjusted and adjusted logistic regression were used to model the associations between the presence/absence of each enteropathogen and elevated inflammation, iron deficiency, and anemia. Anemia was defined as Hb < 110 g/L, per the WHO recommendations [45]. Adjusted models included the child’s sex and age in months, which were chosen *a priori* as potential confounders.  Additional analyses were run to examine effects by relative enteropathogen load (high or low) and by pathogen groupings. High and low pathogen detection were defined as below the median Ct (high) and above the median Ct (low) for each target gene. Adjusted logistic regression models were used to predict inflammation, iron deficiency, and anemia by “high” or “low” relative target gene detection, in reference to no detection. We additionally assessed pathogen detection using three groupings: enteroinvasive bacteria (EIEC/*Shigella*, *C. jejuni/coli*, or *Salmonella*) and potentially enteroinvasive bacteria (EAEC or STEC), non-enteroinvasive, surface adherent bacteria (tEPEC, aEPEC, ST-ETEC, LT-ETEC), or co-detection of both enteroinvasive and non-enteroinvasive bacteria. Logistic regression was also used to evaluate the outcomes comparing detection of enteroinvasive pathogens and co-detection of enteroinvasive and non-enteroinvasive pathogens relative to only non-enteroinvasive pathogens, adjusting for child age and sex. |
|  |  | (*b*) Describe any methods used to examine subgroups and interactions |  | N/A |
|  |  | (*c*) Explain how missing data were addressed | 13 | Of the 265 children’s stool samples that were analyzed for enteropathogens by qPCR, we excluded one sample with missing data on age and two samples lacking biomarker data, for a final analytic sample of 262 children (**Fig 1**). |
|  |  | (*d*) *Cohort study*—If applicable, explain how loss to follow-up was addressed  *Case-control study*—If applicable, explain how matching of cases and controls was addressed  *Cross-sectional study*—If applicable, describe analytical methods taking account of sampling strategy |  | N/A |
|  |  | (*e*) Describe any sensitivity analyses | 12 | As a sensitivity analysis, we used adjusted linear regression to model associations between enteropathogen detection and the outcomes of interest as continuous variables (i.e., CRP, AGP, SF, sTfR, and Hb concentrations). |
| Results |  |  |  |  |
| Participants | 13* | (a) Report numbers of individuals at each stage of study—eg numbers potentially eligible, examined for eligibility, confirmed eligible, included in the study, completing follow-up, and analysed |  | Fig 1, See #6, #10 |
|  |  | (b) Give reasons for non-participation at each stage |  | See #6 |
|  |  | (c) Consider use of a flow diagram |  | Fig 1 |
| Descriptive data | 14* | (a) Give characteristics of study participants (eg demographic, clinical, social) and information on exposures and potential confounders | 13  15 | On average, children were 28 months old, with 47% of children between 6-23 months old and 53% between 24-59 months old (**Table** **1**). Most households had access to an improved drinking water source, but access to adequate sanitation facilities varied, with almost one-third of households practicing open defecation, and less than 3% with a handwashing facility.  Enteropathogens were detected in 87.0% of children’s stool (**Table 3**). One-third of children were positive for one pathogen, one-third for two pathogens, and three or four pathogens were detected in one-fifth of children. EAEC and aEPEC were most common, detected in 59.2% and 46.2% of children’s stool, respectively. Among the other pathogens assessed, 15.3% of stool samples were positive for EIEC/*Shigella*, 13.4% for LT-ETEC, 11.1% for *C. jejuni/coli*, 7.6% for ST-ETEC, 6.5% for tEPEC, and 4.2% for STEC. *Salmonella* was rare (detected in only two children) and *V. cholerae* was not detected. Enteropathogens were found across all sampled age ranges (**Fig 2**). |
|  |  | (b) Indicate number of participants with missing data for each variable of interest | 13,14 | Table 1, Table 2 |
|  |  | (c) *Cohort study*—Summarise follow-up time (eg, average and total amount) |  |  |
| Outcome data | 15* | *Cohort study*—Report numbers of outcome events or summary measures over time |  |  |
|  |  | *Case-control study—*Report numbers in each exposure category, or summary measures of exposure |  |  |
|  |  | *Cross-sectional study—*Report numbers of outcome events or summary measures | 13 | Overall, 45.8% of children were anemic, of whom 46.7% had mild anemia, 50.0% had moderate anemia, and 3.3% had severe anemia (Table 2). One quarter of children had iron deficiency, as defined by low SF concentrations. Sixteen percent of children had elevated CRP concentrations while 37.8% had elevated AGP concentrations. No children had a detectable helminth infection, while 8.4% were positive for malaria parasitemia. |
| Main results | 16 | (*a*) Give unadjusted estimates and, if applicable, confounder-adjusted estimates and their precision (eg, 95% confidence interval). Make clear which confounders were adjusted for and why they were included | 17 | Fig 3, S4 Table |
|  |  | (*b*) Report category boundaries when continuous variables were categorized |  | S4 Table |
|  |  | (*c*) If relevant, consider translating estimates of relative risk into absolute risk for a meaningful time period |  | N/A |
| Other analyses | 17 | Report other analyses done—eg analyses of subgroups and interactions, and sensitivity analyses | 18 | Table 4, S5 Table, S6 Table |
| Discussion |  |  |  |  |
| Key results | 18 | Summarise key results with reference to study objectives | 19 | This cross-sectional study investigated the role of enteropathogens in the etiology of anemia among 6-59-month-old children in semi-rural communities of Greater Accra, Ghana. Anemia affected almost half of children, though less than one-fifth of children had iron deficiency anemia. Almost forty percent of children had elevated CRP or AGP concentrations, indicative of inflammatory responses to infection. These results alongside the finding that CRP and AGP were strongly correlated with Hb concentrations suggest that anemia in this sample is in part attributable to infectious factors, in agreement with recent studies of anemia in Ghanaian children [10,11]. As has been found in other studies investigating the burden of enteropathogens among children in LMICs, most children (87.0%) in this study had at least one bacterial enteropathogen detected in their stool, with EAEC and aEPEC being the most prevalent [20,47]. Enteropathogens presented as predominantly subclinical, with only one in every 15 children experiencing diarrhea. Despite few apparent clinical symptoms, the presence of Campylobacter in stool was strongly associated with systemic inflammation. Interestingly, EAEC detection was associated with anemia, but not with iron deficiency. However, EIEC/Shigella was associated with both iron deficiency and anemia, and children positive for EIEC/Shigella exhibited 5 g/L lower Hb concentrations on average than children without EIEC/Shigella. |
| Limitations | 19 | Discuss limitations of the study, taking into account sources of potential bias or imprecision. Discuss both direction and magnitude of any potential bias | 22,23 | First, we could not ascertain temporal relationships between enteropathogen detection and the outcomes given the cross-sectional study design. Since inflammation may occur days after an infection, with iron deficiency and anemia taking several weeks to develop, concurrent collection of stool and blood samples precluded longitudinal assessment of relationships. Thus, observed associations with anemia can reflect either prior infections or persistent infections, which we did not measure. Furthermore, the high sensitivity of molecular diagnostics means that pathogen detection can represent clinically-relevant infections as well as low-level pathogen exposure and carriage without colonization [43]. Identifying clinically-relevant cut-offs for enteropathogen detection, particularly of asymptomatic infections, remains a work in-progress. Second, the study sample size was smaller than that of many multi-site studies, which may have lowered our power to detect associations, especially among pathogens with a low prevalence, even though our sample size was within the range of per-site sample sizes in the MAL-ED birth cohort [31]. Third, since data were collected in only one region of Ghana and stratified by chicken ownership, enteropathogen prevalence estimates cannot be generalized to all children in this or other regions of Ghana. Finally, children’s nutrient intake may influence their susceptibility to enteropathogenic infections via differences in gut function [31], which we did not measure in our study. Thus, further investigations into the role of diet in modifying associations between infection and anemia are warranted. |
| Interpretation | 20 | Give a cautious overall interpretation of results considering objectives, limitations, multiplicity of analyses, results from similar studies, and other relevant evidence | 23,24 | Our findings suggest that subclinical presence of specific invasive enteropathogenic bacteria, particularly EAEC and EIEC/*Shigella*, may contribute to anemia in young children. Prior literature has found that poor water and sanitation are associated with anemia among young children living in LMICs [48,49], with the hypothesis that exposure to enteropathogens underlies this relationship [14]. By demonstrating an association between certain enteropathogens and anemia, our results lend direct evidence to support this hypothesis. Addressing subclinical enteric infections through improved water, sanitation, and hygiene infrastructure and other approaches such as vaccines should help reduce the anemia burden among young children in Ghana and other similar settings. Additional studies are needed to confirm our findings and assess the relative contribution of enteropathogens to children’s anemia burden, as compared to other infectious diseases and micronutrient deficiencies. Furthermore, research involving longitudinal assessment of individual enteropathogens and iron deficiency and anemia, alongside EED markers, hepcidin, and systemic inflammatory biomarkers, would improve our understanding of the mechanisms linking bacterial enteropathogens and anemia among young children. |
| Generalisability | 21 | Discuss the generalisability (external validity) of the study results |  | See #19 |
| Other information |  |  |  |  |
| Funding | 22 | Give the source of funding and the role of the funders for the present study and, if applicable, for the original study on which the present article is based |  | This research was supported in part by the University of Michigan International Institute, the University of Michigan African Studies Center, the University of Michigan Office of Global Public Health, the University of Michigan Rackham Graduate School, and The Dow Chemical Company Foundation through the Dow Sustainability Fellows Program at the University of Michigan. None of the funders had any role in the study design, data collection, data analysis, data interpretation, or preparation of the manuscript. |

*Give information separately for cases and controls in case-control studies and, if applicable, for exposed and unexposed groups in cohort and cross-sectional studies.

**Note:** An Explanation and Elaboration article discusses each checklist item and gives methodological background and published examples of transparent reporting. The STROBE checklist is best used in conjunction with this article (freely available on the Web sites of PLoS Medicine at http://www.plosmedicine.org/, Annals of Internal Medicine at http://www.annals.org/, and Epidemiology at http://www.epidem.com/). Information on the STROBE Initiative is available at www.strobe-statement.org.
