# Supplementary material for: Associations of bacterial enteropathogens with systemic inflammation, iron deficiency, and anemia in preschool-age children in southern Ghana
Source: PLoS One. 2022 Jul 8;17(7):e0271099. doi: 10.1371/journal.pone.0271099 (PMC9269377; doi:10.1371/journal.pone.0271099)
Supplement: S1 Table — (DOCX) [file pone.0271099.s004.docx]

**S1 Table.** Primer and probe sequences for qPCR detection of pathogen gene targets and assay performance characteristics.^1^

| **Pathogen** | **Target Gene** | **Primer and probe sequences^2^**  **(F: Forward primer, R: Reverse primer, P: Probe)** | **Ref** | **PCR efficiency, %**  **(95% CI)** | **PCR R2** |
| --- | --- | --- | --- | --- | --- |
| *C. jejuni/C. coli* | *cadF* | F: CTGCTAAACCATAGAAATAAAATTTCTCAC  R: CTTTGAAGGTAATTTAGATATGGATAATCG  P: CATTTTGACGATTTTTGGCTTGA | (1) | 88.8 (88.1, 89.5) | 1.000 |
| EPEC | *eae* | F: CATTGATCAGGATTTTTCTGGTGATA  R: CTCATGCGGAAATAGCCGTTA  P: CGAATACTGGCGAGACTATTTCAA | Modified from (1)^3^ | 87.3 (77.3, 100.1) | 0.982 |
| EPEC | *bfpA* | F: TGGTGCTTGCGCTTGCT  R: CGTTGCGCTCATTACTTCTG  P: CAGTCTGCGTCTGATTCCAA | (1) | 107.9 (102.2, 114.3) | 0.997 |
| STEC | *stx1* | F: ACTTCTCGACTGCAAAGACGTATG  R: ACAAATTATCCCCTGWGCCACTATC  P: CTCTGCAATAGGTACTCCA | (1) | 105.2 (96.7, 115.2) | 0.990 |
| STEC | *stx2* | F: CCACATCGGTGTCTGTTATTAACC  R: GGTCAAAACGCGCCTGATAG  P: TTGCTGTGGATATACGAGG | (1) | 112.8 (110.6, 115.0) | 1.000 |
| EAEC | *aatA* | F: CTGGCGAAAGACTGTATCAT  R: TTTTGCTTCATAAGCCGATAGA  P: TGGTTCTCATCTATTACAGACAGC | (1) | 87.6 (85.9, 89.3) | 0.999 |
| EAEC | *aaiC* | F: ATTGTCCTCAGGCATTTCAC  R: ACGACACCCCTGATAAACAA  P: TAGTGCATACTCATCATTTAAG | (1) | 97.3 (96.2, 98.4) | 1.000 |
| LT-ETEC | *LT* | F: TTCCCACCGGATCACCAA  R: CAACCTTGTGGTGCATGATGA  P: CTTGGAGAGAAGAACCCT | (1) | 105.7 (103.7, 107.8) | 0.999 |
| ST-ETEC | *STh* | F: TTCACCTTTCGCTCAGGATG  R: AGCACCCGGTACAAGCAG  P: ATTACTGCTGTGAATTGTG | (2) | 95.6 (94.8, 96.4) | 1.000 |
| ST-ETEC | *STp* | F: TGAATCACTTGACTCTTCAAAA  R: GGCAGGATTACAACAAAGTT  P: TGAACAACACATTTTACTGCT | (1) | 95.0 (89.4, 101.3) | 0.990 |
| EIEC/*Shigella* | *ipaH* | F: CCTTTTCCGCGTTCCTTGA  R: CGGAATCCGGAGGTATTGC  P: CGCCTTTCCGATACCGTCTCTGCA | (1) | 91.9 (91.1, 92.7) | 1.000 |
| *Salmonella enterica* | *ttr* | F: CTCACCAGGAGATTACAACATGG  R: AGCTCAGACCAAAAGTGACCATC  P: CACCGACGGCGAGACCGACTTT | (1) | N/A^4^ | N/A |
| *V. cholerae* | *hlyA* | F: ATCGTCAGTTTGGAGCCAGT  R: TCGATGCGTTAAACACGAAG  P: TCGATGCGTTAAACACGAAG | (1) | N/A^4^ | N/A |

^1^PCR efficiency and R^2^ of the dilution series curve was determined using pooled known positives from child stool samples (≥4 samples) in eight four-fold dilutions with four technical replicates at each dilution. Linear regression of the average Ct against the log10-dilution was used to estimate the slope (β ± 95% confidence interval) and R2 of the standard curve.

Abbreviations: *C. jejuni/coli*, *Campylobacter jejuni* or *Campylobacter coli*; EAEC, enteroaggregative *Escherichia coli (E. coli)*; EIEC, enteroinvasive *E. coli*; EPEC, enteropathogenic *E. coli*; LT-ETEC, heat-labile enterotoxin-producing *E. coli*; ST-ETEC, heat-stable enterotoxin-producing *E. coli*; STEC, Shiga toxin-producing *E. coli*, *V. cholerae*, *Vibrio cholerae;* CI, Confidence Interval; qPCR, quantitative Polymerase Chain Reaction

^2^All probes were double-quencher probes with a 5’ 6-FAM™ fluorophore, internal ZEN™ quencher, and 3’ Iowa Black® Fluorescent Quencher (purchased from Integrated DNA Technologies, IDT).

^3^Base pairs CGA were added to the 5’ end of the probe sequence to increase melting temperature.

^4^An insufficient number of positive samples precluded running a PCR efficiency curve.

References

1. Liu J, Gratz J, Amour C, Nshama R, Walongo T, Maro A, Mduma E, Platts-Mills J, Boisen N, Nataro J, et al. Optimization of quantitative PCR Methods for enteropathogen detection. PLoS One. 2016;11:e0158199. DOI: 10.1371/journal.pone.0158199
2. Taniuchi M, Walters CC, Gratz J, Maro A, Kumburu H, Serichantalergs O, Sethabutr O, Bodhidatta L, Kibiki G, Toney DM, et al. Development of a multiplex polymerase chain reaction assay for diarrheagenic Escherichia coli and Shigella spp. and its evaluation on colonies, culture broths, and stool. Diagn Microbiol Infect Dis; 2012;73:121–8. DOI: 10.1016/j.diagmicrobio.2012.03.008
