# Supplementary material for: Associations of bacterial enteropathogens with systemic inflammation, iron deficiency, and anemia in preschool-age children in southern Ghana
Source: PLoS One. 2022 Jul 8;17(7):e0271099. doi: 10.1371/journal.pone.0271099 (PMC9269377; doi:10.1371/journal.pone.0271099)
Supplement: S2 Table — (DOCX) [file pone.0271099.s005.docx]

S2 Table. Correlation matrix between hemoglobin concentration, iron status biomarkers (SF and sTfR), and inflammatory biomarkers (CRP and AGP) among 262 children aged 6-59 months old in Greater Accra, Ghana.^1^

|  | **Hb** | **SF** | **sTfR** | **CRP** |
| --- | --- | --- | --- | --- |
| **Hb** | - |  |  |  |
| **SF** | **0.363****** | - |  |  |
| **sTfR** | **-0.562****** | **-0.530****** | - |  |
| **CRP** | **-0.226***** | 0.041 | 0.012 | - |
| **AGP** | **-0.187**** | 0.072 | 0.018 | **0.731****** |
| ^1^Values are Pearson correlation coefficients. *p < 0.05, **p < 0.01, ***p < 0.001, ****p < 0.0001. SF, sTfR, CRP, and AGP are natural log-transformed. SF and sTfR are inflammation-adjusted values. Abbreviations: Hb, hemoglobin; SF, serum ferritin; sTfR, serum transferrin receptor; CRP, C-reactive protein; AGP, α-1-acid glycoprotein. | | | | |
