# Supplementary material for: Associations of bacterial enteropathogens with systemic inflammation, iron deficiency, and anemia in preschool-age children in southern Ghana
Source: PLoS One. 2022 Jul 8;17(7):e0271099. doi: 10.1371/journal.pone.0271099 (PMC9269377; doi:10.1371/journal.pone.0271099)
Supplement: S3 Table — (DOCX) [file pone.0271099.s006.docx]

S3 Table. Adjusted associations between enteropathogen detection and symptoms of illness in the past 7 days among children aged 6-59 months old in Greater Accra, Ghana.^1^

| **Pathogen** | **Diarrhea** | **Fever** | **Cough/Cold** | **Nausea** | **Vomiting** |
| --- | --- | --- | --- | --- | --- |
| EAEC | 2.87 (0.79, 10.45) | 0.83 (0.47, 1.48) | 0.74 (0.42, 1.32) | 1.92 (0.59, 6.31) | 1.04 (0.36, 3.01) |
| aEPEC | 0.60 (0.21, 1.69) | 0.89 (0.51, 1.56) | 0.92 (0.52, 1.61) | 1.03 (0.36, 2.92) | 1.18 (0.43, 3.25) |
| LT-ETEC | 1.97 (0.59, 6.52) | 1.67 (0.78, 3.60) | 0.73 (0.30, 1.76) | 0.43 (0.05, 3.42) | 0.87 (0.19, 4.03) |
| EIEC/*Shigella* | 1.27 (0.34, 4.72) | 1.02 (0.47, 2.22) | 1.35 (0.64, 2.85) | 2.23 (0.67, 7.45) | 0.80 (0.17, 3.67) |
| *C. jejuni*/*coli* | - | 1.21 (0.50, 2.93) | 1.41 (0.60, 3.31) | 0.56 (0.07, 4.55) | 0.45 (0.06, 3.62) |
| ST-ETEC | 3.42 (0.84, 13.90) | 0.15 (0.02, 1.15) | 0.98 (0.34, 2.85) | 0.97 (0.12, 7.99) | 0.89 (0.11, 7.24) |
| tEPEC | 1.68 (0.34, 8.27) | 1.77 (0.62, 5.04) | 0.62 (0.17, 2.24) | 2.46 (0.50, 12.10) | 2.05 (0.42, 10.00) |
| STEC | **5.78* (1.02, 32.64)** | 0.61 (0.13, 2.95) | 0.29 (0.04, 2.36) | - | - |
| ^1^Values are Odds Ratios (95% Confidence Intervals) using logistic regression models, adjusting for child sex and age in months. **p < 0.05, **p < 0.01.* Dash (-) indicates that the logistic model could not be run due to complete separation. Sample size: n=262  Abbreviations: aEPEC, atypical enteropathogenic *Escherichia coli (E. coli)*; *C. jejuni/coli*, *Campylobacter jejuni* or *Campylobacter coli*; EAEC, enteroaggregative *E. coli*; EIEC, enteroinvasive *E. coli*; LT-ETEC, heat-labile enterotoxin-producing *E. coli*; STEC, Shiga toxin-producing *E. coli;* ST-ETEC, heat-stable enterotoxin-producing *E. coli;* tEPEC, typical enteropathogenic *E. coli.* | | | | | |
