# Supplementary material for: Associations of bacterial enteropathogens with systemic inflammation, iron deficiency, and anemia in preschool-age children in southern Ghana
Source: PLoS One. 2022 Jul 8;17(7):e0271099. doi: 10.1371/journal.pone.0271099 (PMC9269377; doi:10.1371/journal.pone.0271099)
Supplement: S4 Table — (DOCX) [file pone.0271099.s007.docx]

S4 Table. Adjusted associations between enteropathogen detection and elevated systemic inflammation, iron deficiency, and anemia among 262 Ghanaian children aged 6-59 months old.^1^

| **Pathogen** | **Inflammation** | |  | **Iron Deficiency** | |  | **Anemia** |
| --- | --- | --- | --- | --- | --- | --- | --- |
|  | **CRP > 5mg/L** | **AGP > 1g/L** |  | **SF < 12 ug/L** | **sTfR > 8.3 mg/L** |  | **Hb < 110 g/L** |
| EAEC | 0.57 (0.29, 1.11) | 0.91 (0.54, 1.52) |  | 0.84 (0.46, 1.53) | 0.82 (0.49, 1.36) |  | **1.69* (1.01, 2.84)** |
| aEPEC | **0.44* (0.22, 0.89)** | **0.48** (0.29, 0.81)** |  | 0.73 (0.40, 1.30) | 0.70 (0.42, 1.15) |  | 0.81 (0.49, 1.33) |
| LT-ETEC | 0.28 (0.07, 1.24) | 0.76 (0.35, 1.63) |  | 0.54 (0.21, 1.39) | 0.58 (0.27, 1.23) |  | 0.91 (0.44, 1.89) |
| EIEC/*Shigella* | 1.87 (0.83, 4.20) | 1.26 (0.63, 2.50) |  | **2.55* (1.23, 5.29)** | 1.30 (0.66, 2.57) |  | **2.34* (1.15, 4.76)** |
| *C. jejuni/coli* | **3.49** (1.45, 8.41)** | **4.27** (1.85, 9.84)** |  | 1.68 (0.73, 3.86) | 1.67 (0.75, 3.70) |  | 1.29 (0.58, 2.86) |
| ST-ETEC | 0.48 (0.11, 2.16) | 0.63 (0.23, 1.72) |  | 1.10 (0.37, 3.28) | 0.65 (0.24, 1.72) |  | 0.53 (0.19, 1.47) |
| tEPEC | 1.69 (0.52, 5.53) | 1.26 (0.46, 3.46) |  | 0.76 (0.23, 2.51) | 0.58 (0.20, 1.64) |  | 1.19 (0.44, 3.25) |
| STEC | 0.46 (0.06, 3.74) | 2.72 (0.76, 9.67) |  | 1.68 (0.41, 6.97) | 1.96 (0.56, 6.87) |  | 1.29 (0.37, 4.54) |
| ^1^Values are Odds Ratios (95% Confidence Intervals) using logistic regression models, adjusting for child sex and age in months. **p < 0.05, **p < 0.01.*  Abbreviations: aEPEC, atypical enteropathogenic *Escherichia coli (E. coli)*; AGP, α-1-acid glycoprotein; *C. jejuni/coli*, *Campylobacter jejuni* or *Campylobacter coli*; CRP, C-reactive protein; EAEC, enteroaggregative *E. coli*; EIEC, enteroinvasive *E. coli*; Hb, hemoglobin; LT-ETEC, heat-labile enterotoxin-producing *E. coli*; SF, serum ferritin; STEC, Shiga toxin-producing *E. coli;* ST-ETEC, heat-stable enterotoxin-producing *E. coli;* sTfR, serum transferrin receptor; tEPEC, typical enteropathogenic *E. coli.* | | | | | | | |
