# Supplementary material for: Associations of bacterial enteropathogens with systemic inflammation, iron deficiency, and anemia in preschool-age children in southern Ghana
Source: PLoS One. 2022 Jul 8;17(7):e0271099. doi: 10.1371/journal.pone.0271099 (PMC9269377; doi:10.1371/journal.pone.0271099)
Supplement: S5 Table — (DOCX) [file pone.0271099.s008.docx]

S5 Table. Adjusted associations of high and low relative gene target quantity and inflammation (CRP and AGP), iron deficiency (SF and sTfR), and anemia (Hb) among children aged 6-59 months old in Greater Accra, Ghana.^1^

| **Pathogen** | **Gene Target** | **Relative Quantity** | **N** | **CRP > 5 mg/L** | **AGP > 1 g/L** | **SF < 12 µg/L** | **sTfR > 8.3 mg/L** | **Hb < 110 g/L** |
| --- | --- | --- | --- | --- | --- | --- | --- | --- |
| EIEC/*Shigella* | *ipaH* | No detection | 222 | Ref | Ref | Ref | Ref | Ref |
|  |  | Low (Ct ≥ 29.8) | 21 | 0.94 (0.26, 3.40) | 0.68 (0.25, 1.82) | **2.97* (1.15, 7.69)** | 2.18 (0.86, 5.52) | **2.83* (1.08, 7.45)** |
|  |  | High (Ct < 29.8) | 19 | **3.24* (1.18, 8.89)** | 2.36 (0.91, 6.15) | 2.14 (0.78, 5.90) | 0.73 (0.27, 1.94) | 1.91 (0.73, 5.05) |
| *C. jejuni/coli* | *cadF* | No detection | 233 | Ref | Ref | Ref | Ref | Ref |
|  |  | Low (Ct ≥ 28.4) | 14 | **3.74* (1.16, 12.10)** | **3.92* (1.25, 12.27)** | 1.20 (0.36, 4.06) | 1.68 (0.56, 5.03) | 1.44 (0.48, 4.38) |
|  |  | High (Ct < 28.4) | 15 | **3.27* (1.02, 10.55)** | **4.64** (1.49, 14.43)** | 2.24 (0.75, 6.68) | 1.66 (0.56, 4.91) | 1.15 (0.39, 3.40) |
| EAEC | *aatA* | No detection | 122 | Ref | Ref | Ref | Ref | Ref |
|  |  | Low (Ct ≥ 28.3) | 70 | 0.49 (0.21, 1.17) | 0.74 (0.40, 1.38) | 0.82 (0.39, 1.73) | 0.84 (0.46, 1.54) | 1.44 (0.79, 2.64) |
|  |  | High (Ct < 28.3) | 70 | 0.59 (0.25, 1.38) | 1.09 (0.58, 2.05) | 1.14 (0.57, 2.30) | 0.77 (0.41, 1.43) | **1.88* (1.00, 3.51)** |
| EAEC | *aaiC* | No detection | 184 | Ref | Ref | Ref | Ref | Ref |
|  |  | Low (Ct ≥ 26.3) | 39 | 0.98 (0.37, 2.57) | 1.11 (0.54, 2.31) | 0.82 (0.35, 1.89) | 1.39 (0.68, 2.80) | 1.71 (0.83, 3.49) |
|  |  | High (Ct < 26.3) | 39 | 1.18 (0.47, 2.95) | **2.07* (1.02, 4.19)** | 1.14 (0.51, 2.53) | 0.76 (0.37, 1.57) | 1.04 (0.51, 2.11) |
| tEPEC/ aEPEC/ STEC | *eae* | No detection | 99 | Ref | Ref | Ref | Ref | Ref |
|  |  | Low (Ct ≥ 29.0) | 80 | **0.38* (0.17, 0.88)** | 0.60 (0.32, 1.11) | 0.66 (0.32, 1.36) | 0.82 (0.45, 1.49) | 1.00 (0.54, 1.83) |
|  |  | High (Ct < 29.0) | 83 | **0.43* (0.19, 0.97)** | 0.77 (0.42, 1.41) | 0.83 (0.42, 1.64) | 0.78 (0.43, 1.42) | 0.70 (0.38, 1.28) |
| tEPEC | *bfpA* | No detection | 240 | Ref | Ref | Ref | Ref | Ref |
|  |  | Low (Ct ≥ 25.8) | 11 | 1.26 (0.26, 6.14) | 1.05 (0.30, 3.72) | 2.35 (0.66, 8.35) | 1.37 (0.40, 4.74) | 1.28 (0.37, 4.38) |
|  |  | High (Ct < 25.8) | 11 | 2.14 (0.53, 8.67) | 2.39 (0.69, 8.21) | 0.21 (0.03, 1.74) | 0.36 (0.09, 1.42) | 0.77 (0.22, 2.67) |
| STEC | *stx1* | No detection | 245 | Ref | Ref | Ref | Ref | Ref |
|  |  | Low (Ct ≥ 32.4) | 9 | 0.58 (0.07, 4.81) | 3.39 (0.82, 14.05) | 3.14 (0.76, 13.02) | 0.67 (0.16, 2.80) | 0.35 (0.07, 1.76) |
|  |  | High (Ct < 32.4) | 8 | - | 1.97 (0.48, 8.13) | 0.92 (0.18, 4.72) | 1.92 (0.44, 8.31) | 1.61 (0.37, 6.95) |
| STEC | *stx2* | No detection | 249 | Ref | Ref | Ref | Ref | Ref |
|  |  | Low (Ct ≥ 32.1) | 6 | 2.29 (0.40, 13.19) | 8.17 (0.93, 71.65) | 4.78 (0.83, 27.46) | 8.10 (0.89, 73.77) | 8.87 (0.96, 81.68) |
|  |  | High (Ct < 32.1) | 7 | - | 4.24 (0.80, 22.51) | 1.58 (0.28, 9.05) | 0.23 (0.03, 1.95) | 0.55 (0.10, 3.00) |
| LT-ETEC/ ST-ETEC | *LT* | No detection | 215 | Ref | Ref | Ref | Ref | Ref |
|  |  | Low (Ct ≥ 29.2) | 23 | 0.19 (0.03, 1.48) | 0.85 (0.34, 2.11) | 0.69 (0.24, 2.00) | 0.45 (0.18, 1.17) | 0.96 (0.40, 2.32) |
|  |  | High (Ct < 29.2) | 24 | 0.19 (0.02, 1.43) | 0.53 (0.20, 1.40) | 0.35 (0.10, 1.25) | 0.63 (0.26, 1.54) | 0.63 (0.26, 1.52) |
| ST-ETEC | *STh* | No detection | 253 | Ref | Ref | Ref | Ref | Ref |
|  |  | Low (Ct ≥ 21.8) | 4 | - | 0.44 (0.04, 4.40) | - | 1.34 (0.17, 10.43) | 0.49 (0.05, 5.21) |
|  |  | High (Ct < 21.8) | 5 | 3.41 (0.54, 21.39) | 0.41 (0.04, 3.73) | 4.65 (0.73, 29.43) | 0.28 (0.03, 2.60) | 0.27 (0.03, 2.47) |
| ST-ETEC | *STp* | No detection | 250 | Ref | Ref | Ref | Ref | Ref |
|  |  | Low (Ct ≥ 30.2) | 6 | - | 0.74 (0.13, 4.18) | - | 0.63 (0.11, 3.60) | 0.27 (0.03, 2.43) |
|  |  | High (Ct < 30.2) | 6 | - | 1.52 (0.30, 7.79) | 1.84 (0.31, 11.01) | 0.66 (0.12, 3.74) | 1.39 (0.26, 7.35) |
| ^1^Values are Odds Ratio (95% Confidence Interval) using logistic regression models, adjusting for child sex and age in months. High- and low-relative quantity are defined as below and above the median Ct for each gene target, excluding detection of Ct > 35. **p < 0.05, **p < 0.01.* SF and sTfR cut-offs use inflammation-adjusted values. Sample size: n=262  Abbreviations: aEPEC, atypical enteropathogenic *Escherichia coli (E. coli)*; AGP, α-1-acid glycoprotein; *C. jejuni/coli*, *Campylobacter jejuni* or *Campylobacter coli*; CRP, C-reactive protein; EAEC, enteroaggregative *E. coli*; EIEC, enteroinvasive *E. coli*; Hb, hemoglobin; LT-ETEC, heat-labile enterotoxin-producing *E. coli*; SF, serum ferritin; STEC, Shiga toxin-producing *E. coli;* ST-ETEC, heat-stable enterotoxin-producing *E. coli;* sTfR, serum transferrin receptor; tEPEC, typical enteropathogenic *E. coli.* | | | | | | | | |
