# Supplementary material for: Associations of bacterial enteropathogens with systemic inflammation, iron deficiency, and anemia in preschool-age children in southern Ghana
Source: PLoS One. 2022 Jul 8;17(7):e0271099. doi: 10.1371/journal.pone.0271099 (PMC9269377; doi:10.1371/journal.pone.0271099)
Supplement: S6 Table — (DOCX) [file pone.0271099.s009.docx]

S6 Table. Adjusted associations between enteropathogen detection and concentrations of inflammatory biomarkers (CRP and AGP), iron status biomarkers (SF and sTfR), and hemoglobin (Hb) among children aged 6-59 months old in Greater Accra, Ghana.^1^

| **Pathogen** | **CRP (mg/L)** | **AGP (g/L)** | **SF (µg/L)** | **sTfR (mg/L)** | **Hb (g/L)** |
| --- | --- | --- | --- | --- | --- |
| EAEC | -1.39 (-4.27, 1.49) | -0.09 (-0.23, 0.05) | -2.78 (-8.59, 3.03) | 0.13 (-1.17, 1.43) | -2 (-6, 1) |
| aEPEC | -1.79 (-4.58, 1.00) | **-0.23** (-0.36, -0.10)** | -1.61 (-7.25, 4.02) | -0.89 (-2.15, 0.36) | 3 (-1, 6) |
| LT-ETEC | -3.23 (-7.32, 0.87) | -0.16 (-0.36, 0.04) | 0.25 (-8.04, 8.53) | -0.39 (-2.24, 1.46) | 2 (-3, 7) |
| EIEC/*Shigella* | 0.86 (-3.02, 4.74) | 0.04 (-0.15, 0.23) | -5.52 (-13.32, 2.28) | 1.36 (-0.38, 3.10) | **-5* (-10, -0.3)** |
| *C. jejuni/coli* | 3.26 (-1.23, 7.74) | **0.34** (0.12, 0.55)** | -0.78 (-9.85, 8.29) | 1.04 (-0.98, 3.06) | -3 (-9, 3) |
| ST-ETEC | -3.37 (-8.66, 1.92) | -0.20 (-0.45, 0.06) | 1.55 (-9.14, 12.24) | -1.72 (-4.10, 0.65) | 5 (-2, 12) |
| tEPEC | **7.50** (1.89, 13.12)** | 0.19 (-0.09, 0.47) | 1.34 (-10.12, 12.81) | -1.65 (-4.20, 0.90) | -2 (-9, 6) |
| STEC | -2.86 (-9.87, 4.15) | 0.09 (-0.25, 0.43) | -10.99 (-25.07, 3.09) | 2.67 (-0.47, 5.81) | 0.4 (-8, 9) |
| ^1^Values are β coefficients (95% Confidence Intervals) from linear regression models, adjusting for child sex and age in months. SF and sTfR are inflammation-adjusted values. **p < 0.05, **p < 0.01* Sample size: n=262  Abbreviations: aEPEC, atypical enteropathogenic *Escherichia coli (E. coli)*; AGP, α-1-acid glycoprotein; *C. jejuni/coli*, *Campylobacter jejuni* or *Campylobacter coli*; CRP, C-reactive protein; EAEC, enteroaggregative *E. coli*; EIEC, enteroinvasive *E. coli*; Hb, hemoglobin; LT-ETEC, heat-labile enterotoxin-producing *E. coli*; SF, serum ferritin; STEC, Shiga toxin-producing *E. coli;* ST-ETEC, heat-stable enterotoxin-producing *E. coli;* sTfR, serum transferrin receptor; tEPEC, typical enteropathogenic *E. coli.* | | | | | |
